# MAPS: Model-based analysis of long-range chromatin interactions from PLAC-seq and HiChIP experiments

**DOI:** 10.1371/journal.pcbi.1006982

**Published:** 2019-04-15

**Authors:** Ivan Juric, Miao Yu, Armen Abnousi, Ramya Raviram, Rongxin Fang, Yuan Zhao, Yanxiao Zhang, Yunjiang Qiu, Yuchen Yang, Yun Li, Bing Ren, Ming Hu

**Affiliations:** 1 Department of Quantitative Health Sciences, Lerner Research Institute, Cleveland Clinic Foundation, Cleveland, OH, United States of America; 2 Ludwig Institute for Cancer Research, La Jolla, CA, United States of America; 3 Bioinformatics and Systems Biology Graduate Program, University of California, San Diego, La Jolla, CA, United States of America; 4 Departments of Genetics, Biostatistics, and Computer Science, University of North Carolina, Chapel Hill, NC, United States of America; 5 Department of Cellular and Molecular Medicine, Center for Epigenomics, University of California, San Diego School of Medicine, La Jolla, CA, United States of America; National Cancer Institute, United States of America and Tel Aviv University, Israel, UNITED STATES

## Abstract

Hi-C and chromatin immunoprecipitation (ChIP) have been combined to identify long-range chromatin interactions genome-wide at reduced cost and enhanced resolution, but extracting information from the resulting datasets has been challenging. Here we describe a computational method, MAPS, **M**odel-based **A**nalysis of **P**LAC-**s**eq and HiChIP, to process the data from such experiments and identify long-range chromatin interactions. MAPS adopts a zero-truncated Poisson regression framework to explicitly remove systematic biases in the PLAC-seq and HiChIP datasets, and then uses the normalized chromatin contact frequencies to identify significant chromatin interactions anchored at genomic regions bound by the protein of interest. MAPS shows superior performance over existing software tools in the analysis of chromatin interactions from multiple PLAC-seq and HiChIP datasets centered on different transcriptional factors and histone marks. MAPS is freely available at https://github.com/ijuric/MAPS.

## Author summary

Chromatin spatial organization plays an important role in genome function. The recently developed PLAC-seq and HiChIP technologies have become powerful tools to study long-range chromatin interactions. However, the biases introduced by the ChIP procedure have added substantial difficulty in data analysis. Existing methods all suffer from suboptimal performance. Here we present a new method, named MAPS, to explicitly remove biases in PLAC-seq and HiChIP data and identify long-range chromatin interactions with high reproducibility and accuracy. We benchmark the performance of MAPS using two public datasets and two in-house datasets, and demonstrate that MAPS is superior to existing methods. More importantly, MAPS can identify a large number of biologically relevant chromatin interactions that are missed by state-of-the-art mapping tools.

## Introduction

While millions of candidate enhancers have been predicted in the human genome, annotation of their target genes remains challenging, because enhancers do not always regulate the closest gene in the linear genome sequence [1]. Recognizing that distal enhancers frequently form chromatin loops with the target gene promoters to regulate their expression, evidence of long-range chromatin interactions between enhancers and promoters has been increasingly used to predict the target genes of enhancers and dissect gene regulatory networks [2]. Chromosome conformation capture (3C) [3] based methods, such as in situ Hi-C [4], have been used to detect long-range chromatin interactions in mammalian cells. However, billions of reads are typically needed to achieve kilobase (Kb) resolution, limiting their applications. PLAC-seq [5] and HiChIP [6] technologies combine in situ Hi-C and chromatin immunoprecipitation (ChIP) to efficiently capture chromatin interactions anchored at genomic regions bound by specific proteins or histone modifications, achieving Kb resolution with fewer sequencing reads and much reduced sequencing cost [7] (**Note 1 in S1 Text**).

Several software tools, including Fit-Hi-C [8], HiCCUPS [4], Mango [9] and hichipper [10] have been used to identify long-range chromatin interactions from PLAC-seq and HiChIP data. However, most of these methods are not optimal since they do not take into account PLAC-seq/HiChIP-specific biases. PLAC-seq/HiChIP datasets not only suffer from the biases introduced by differential effective fragment length, GC content and sequence uniqueness that are common to all 3C based methods [11], but also contain the biases introduced during the ChIP procedure (i.e., ChIP enrichment level). For example, Fit-Hi-C and HiCCUPS, developed mainly for Hi-C datasets, utilize the matrix-balancing-based normalization approaches (ICE, VC or KR) [4, 8] to correct the biases in Hi-C data. However, the underlying assumption of these normalization approaches that all genomic regions have equal visibility is invalid for PLAC-seq/HiChIP data since not all the genomic regions are bound by the protein of interest and can be enriched by ChIP. Moreover, the protein-binding regions may be enriched at different levels and such bias in ChIP enrichment level must be taken into consideration. Mango is designed for ChIA-PET which only detects long-range interactions between two genomic regions both bound by the protein of interest. In Mango, MACS2 [12] is first used to call protein binding sites from the data and these 1D peaks are defined as anchor regions to identity long-range interactions (only interactions between two anchor regions are considered). Although Mango considers the ChIP-introduced biases, application of Mango to PLAC-seq/HiChIP data is still problematic for two reasons: 1) PLAC-seq/HiChIP enables detection of valid chromatin interactions between protein-bound regions and non-binding regions, which are not considered by Mango; 2) even for the detection of long-range interactions between two protein-bound regions, Mango is suboptimal since the anchor regions defined by MACS2 suffer from high false positive rate due to PLAC-seq/HiChIP-specific bias. To solve the second problem of Mango, hichipper [10] introduces a bias-corrected peak calling algorithm. However, hichipper still relies on the statistical model in Mango to identify long-range interactions and thus is not designed to call interactions between protein binding regions and non-binding regions (more discussions in **Note 2 in S1 Text**).

To address the aforementioned limitations, we introduce MAPS as a PLAC-seq/HiChIP-specific analysis pipeline. MAPS models the expected contact frequency of pairs of loci accounting for common biases of 3C methods, the PLAC-seq/HiChIP-specific biases and genomic distance effects, and uses this model to determine statistically significant long-range chromatin interactions. In addition, MAPS is able to identify the long-range interactions with both ends bound by the protein of interest as well as the interactions with only one end bound by the protein of interest.

## Results

### Framework of MAPS

**Fig 1:**
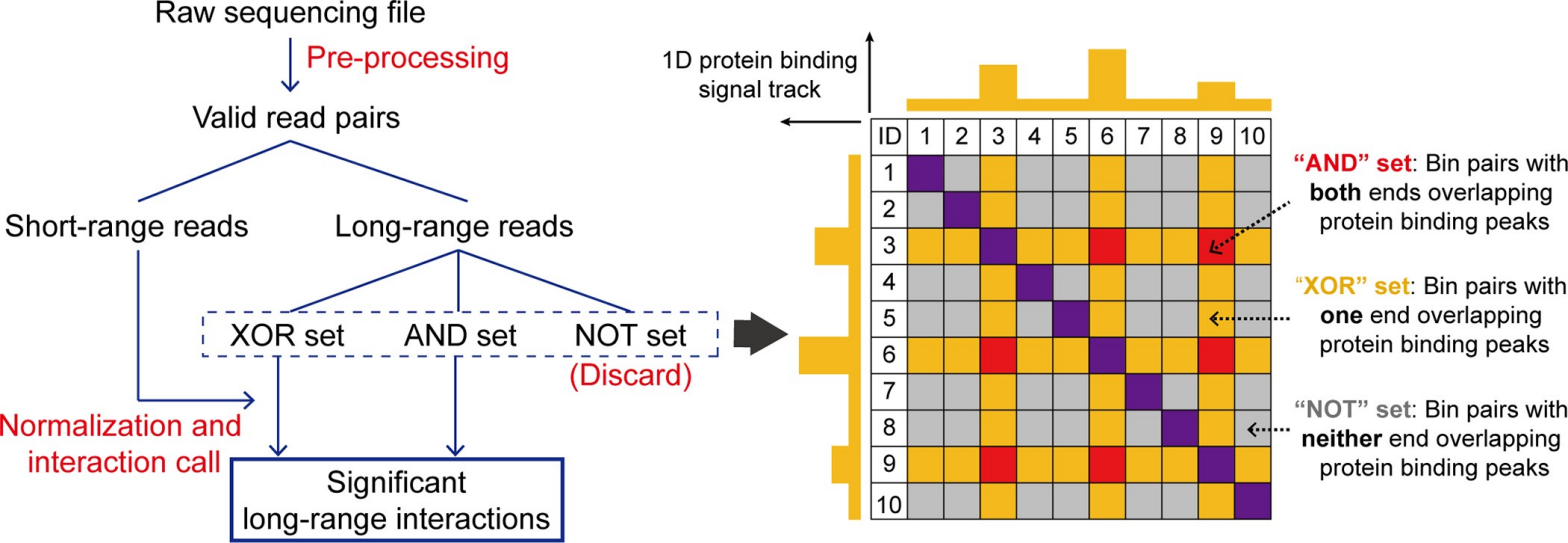
Framework of MAPS. MAPS workflow contains three major components: pre-processing, normalization and long-range interaction determination. Valid read pairs are obtained after pre-processing and then grouped to short-range reads or long-range reads depending on the genomic distance between two ends (1Kb is used). The long-range reads are further classified into the “AND”, “XOR” or “NOT” set. MAPS only considers the “AND” and “XOR” set of bin pairs for normalization and identification of the statistically significant long-range chromatin interactions. Short-range reads are used to estimate and correct for biases introduced by the ChIP procedure.

MAPS workflow contains three major components: pre-processing, normalization and long-range interaction determination (**Fig 1**). In pre-processing, MAPS first takes raw fastq files as input and maps them to the reference genome. Low mapping quality reads, invalid pairs of alignments and PCR duplicates are then removed sequentially to keep valid read pairs. These valid read pairs can be divided into two groups: short-range reads (< = 1Kb) and long-range reads (>1Kb). Since the insert size of PLAC-seq/HiChIP libraries are often less than 1Kb, the majority of short-range reads are dangling ends or self-ligation products of undigested DNA, therefore are free of noise introduced by proximity ligation and can be used to measure ChIP enrichment level for later bias correction (for details see **Methods** and **S1 Fig**). Each long-range read is further assigned into the “NOT”, “XOR” or “AND” set of bin pairs in the contact matrix. As illustrated in **Fig 1**, the “NOT” set refers to bin pairs with neither ends overlapping protein binding peaks; the “XOR” set refers to bin pairs with only one end overlapping protein binding peaks; whereas the “AND” set refers to bin pairs with both ends overlapping protein binding peaks. In this step, MAPS requires a list of protein binding sites as input to define the “NOT”, “XOR” or “AND” set of bin pairs. For the best result, we recommend using the ChIP-seq peak list of the same protein assessed in PLAC-seq/HiChIP experiment from the same cell type. If such list is not available, hichipper can be applied to PLAC-seq/HiChIP data to call protein binding peaks. In the subsequent normalization and interaction calling analysis, only bin pairs in the “AND” and “XOR” sets are considered, since bin pairs in the “NOT” set are not the ChIP targets and often contain fewer reads for reliable interaction call (**S2 Fig** and **S1 Table**).

After pre-processing, MAPS implements a novel statistical model to remove systematic biases in PLAC-seq/HiChIP data. Our group previously developed HiCNorm [13], a method free of the equal-visibility assumption, to remove systematic biases (e.g., effective fragment length, GC content and sequence uniqueness) in Hi-C data. In MAPS, we extended the HiCNorm statistical framework to PLAC-seq/HiChIP data by further incorporating the ChIP enrichment level and the linear genomic distance (see **Methods** and **Note 3 in S1 Text**). As illustrated in **S3 Fig**, the Pearson correlation coefficients between contact frequency and each bias factor are greatly reduced after MAPS normalization. MAPS then calculates the expected contact frequency, P-value and false discovery rate (FDR) of each bin pair in “XOR” and “AND” sets so that significant interactions can be determined with a user-defined FDR threshold. Noticeably, MAPS treats “XOR” or “AND” sets as two independent groups for data normalization, since bin pairs in the “AND” set have much higher contact frequency than bin pairs in the “XOR” set due to the ChIP enrichment on both ends (**S2 Fig** and **S1 Table**). Ignoring the difference in contact frequency between “AND” and “XOR” sets of bin pairs and fitting them with the same model will lead to either under-estimation of background for the “AND” set or over-estimation of background for the “XOR” set.

### Comparison of MAPS with hichipper on four PLAC-seq/HiChIP datasets

To evaluate the performance of MAPS, we applied both MAPS and hichipper to two published HiChIP datasets targeting H3K27ac and Smc1a in GM12878 cells [6, 7] and two in-house PLAC-seq datasets targeting H3K4me3 and CTCF in mouse embryonic stem cells (mESCs) (**S2 Table**). We did not compare MAPS with HiCCUPS or Fit-Hi-C, since both methods are not designed for PLAC-seq/HiChIP, and Lareau and Aryee study [10] has demonstrated that hichipper has higher sensitivity than HiCCUPS, and has better power to detect long-range interactions than Fit-Hi-C.

Considering the sequencing depth of each dataset, we used 10Kb resolution for mESC CTCF PLAC-seq data and 5Kb resolution for the other 3 datasets for interaction calling (see **Note 4 in S1 Text** for results with finer resolution). To minimize false positives and reduce computational burden, we only considered the intra-chromosomal bin pairs (from 2*resolution to 1Mb) in “XOR” and “AND” sets (see **Note 5 in S1 Text** for results with extended genomic range). We defined a tested bin pair as statistically significant if it satisfies the following three criteria simultaneously: 1) FDR < 0.01; 2) normalized contact frequency (i.e., raw read counts/expected read counts) > = 2; 3) raw read counts > = 12. Details of justification of such thresholds can be found in **Note 6 in S1 Text**. We then grouped these significant bin pairs into singletons or clusters, depending on whether additional significant bin pairs exist within their neighborhoods (see **Methods** for details). Since singletons are more likely to be false positives than clusters [4], we applied additional filtering and only kept singletons with FDR < 10^−4^ as significant interactions. To make a fair comparison, the same thresholds described above was used to define significant interactions from hichipper output (see **Methods** for details). We first examined the reproducibility of MAPS and hichipper between two biological replicates: the reproducibility of MAPS calls among biological replicates ranges 69.4% ~ 90.7% for these four datasets, comparable to the results from hichipper (52.1% ~ 92.8%) (**S3 Table**). As a reference, the widely used HiCCUPS for in situ Hi-C data has 64.3% ~ 67.4% reproducibility between biological replicates (**Note 7 in S1 Text**). Since both MAPS- and hichipper-identified interactions are reproducible, we combined the biological replicates and called interactions from the combined data for all downstream analysis.

MAPS identified 37,951, 170,630, 53,788 and 134,179 significant interactions from GM12878 Smc1a, GM12878 H3K27ac, mESC CTCF, and mESC H3K4me3 data, respectively. The application of hichipper resulted in fewer significant interactions (17,982, 113,070, 22,153 and 62,652), which is expected since hichipper does not identify the interactions from the “XOR” set (**S4 Table**). The median distance of the MAPS-identified interactions is larger than that of hichipper-identified interactions (**S4 Fig**) and MAPS detects more >50Kb interactions than hichipper (90.0%~97.1% vs 67.1%~76.6%).

**Fig 2:**
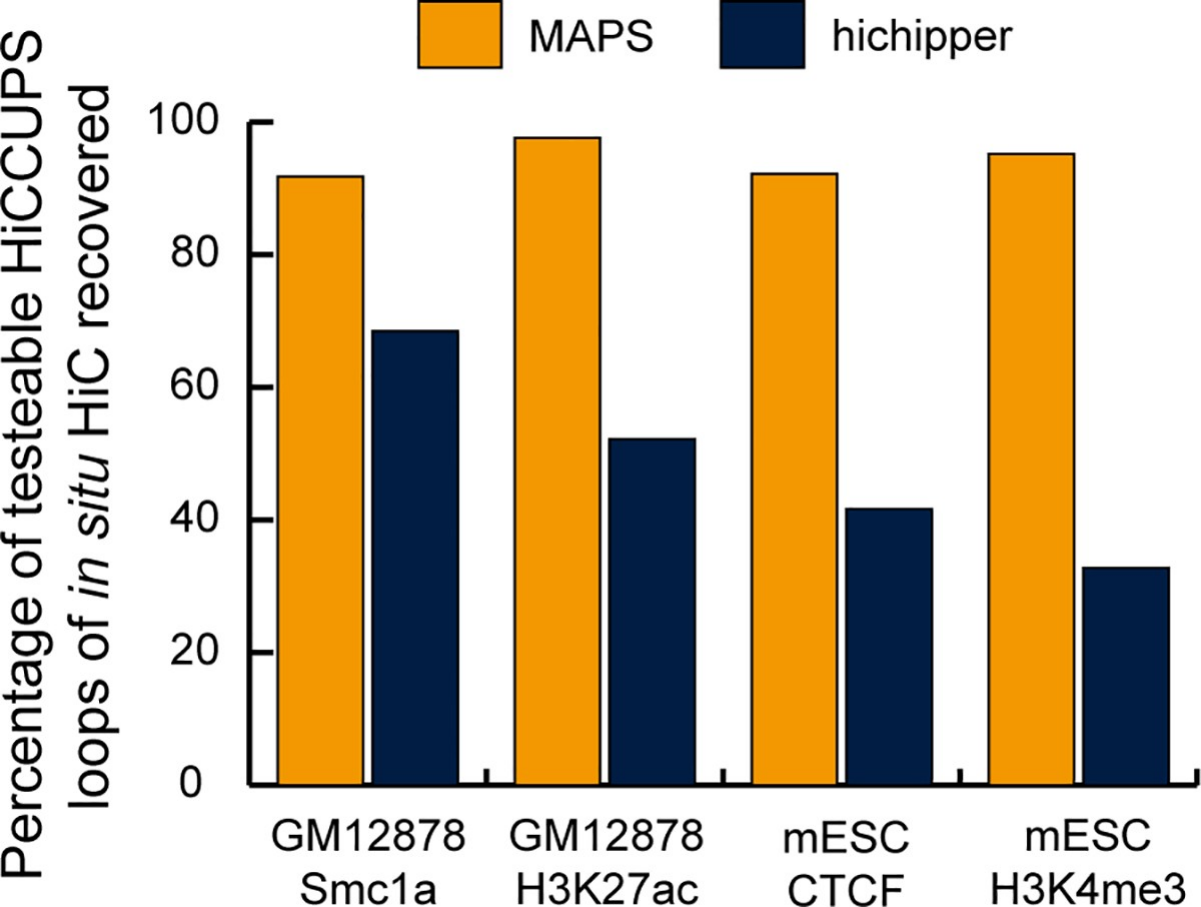
Comparison of sensitivity of MAPS and hichipper. The Y-axis is the sensitivity, defined as the percentage of detectable HiCCUPS loops of deeply sequenced in situ Hi-C data (**S6 Table**) recovered by MAPS- or hichipper-identified interactions.

Next, we compared the sensitivity of MAPS and hichipper in detecting known interactions — the chromatin loops identified by HiCCUPS from deeply sequenced in situ Hi-C data from matching cell types [4, 14] (**S5 Table**). Since PLAC-seq/HiChIP are designed to detect interactions associated with a specific protein, we filtered the chromatin loops from in situ Hi-C and only kept the ones associated with the protein of interest for this analysis (**Note 8 in S1 Text** and **S6 Table**). In all four datasets, MAPS achieved consistently higher sensitivity than hichipper (91.8%, 97.6%, 92.2%, 95.2% vs 68.5%, 52.2%, 41.7%, 32.8%, **Fig 2**). The substantially improved sensitivity of MAPS is largely contributed by its ability to identify interactions from the “XOR” set (**S7 Table** and **Methods**). The benefit of including the “XOR” set for interaction calling is more pronounced for the dataset targeting H3K4me3 compared to the ones targeting CTCF/Smc1a/H3K27ac, since 45.4% - 64.2% HiCCUPS loops belong to the “AND” set when CTCF/Smc1a/H3K27ac is the target protein whereas the proportion drops to only 25.8% when H3K4me3 is the target protein (**S6 Table**).

**Fig 3:**
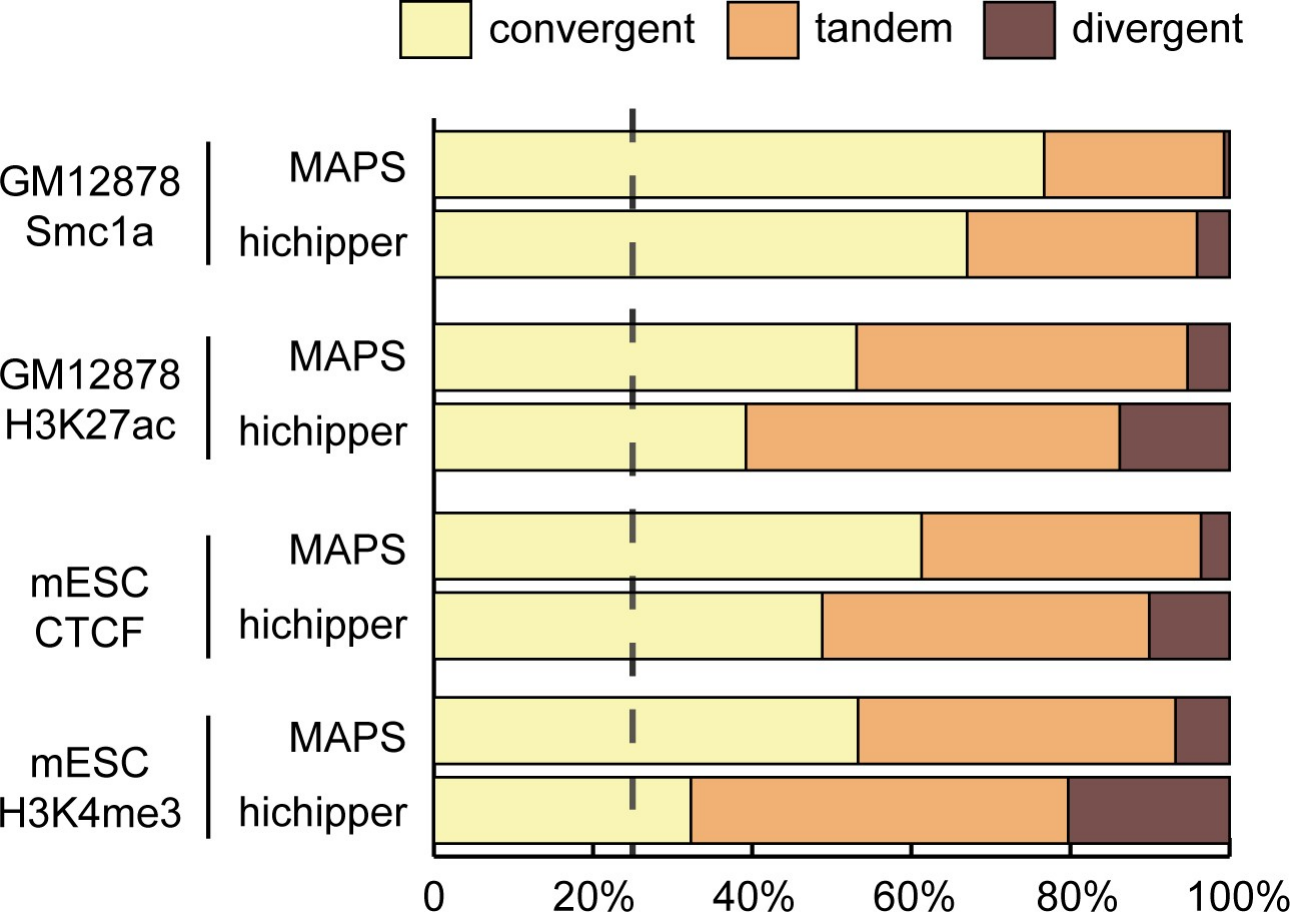
CTCF motif orientation of MAPS- and hichipper-identified interactions. The proportion of convergent, tandem and divergent CTCF motif pairs among testable MAPS- and hichipper-identified interactions. Only interactions with both ends containing either single CTCF motif or multiple CTCF motifs in the same direction are considered. The dotted vertical line indicates the expected convergent proportion from randomly chosen CTCF motif pairs (25%).

We also tried to assess the true positive rate for MAPS- and hichipper-identified interactions. However, due to the lack of a complete list of true interactions in these cells, we instead asked which method may better recapitulate the known feature of chromatin interactions. It is known that CTCF/cohesin-associated interactions have a preference in CTCF motif orientation: 64.5% and 92% of interactions identified from the previous ChIA-PET and in situ Hi-C studies contain convergent CTCF motifs [4, 15]. We checked the CTCF motif orientation of the testable MAPS-identified interactions and found the convergent CTCF motif rate is 76.7%, 53.1%, 61.3% and 53.3% for GM12878 Smc1a, GM12878 H3K27ac, mESC CTCF, and mESC H3K4me3 data, respectively (see **Methods** for details). By comparison, convergent CTCF motif is found with lower proportions in the testable hichipper-identified interactions (67.0%, 39.2%, 48.8% and 32.3%, Chi-square test p-values <2.2e-16 for all four datasets), suggesting MAPS yielded more accurate interaction calls than hichipper (**Fig 3**).

**Fig 4:**
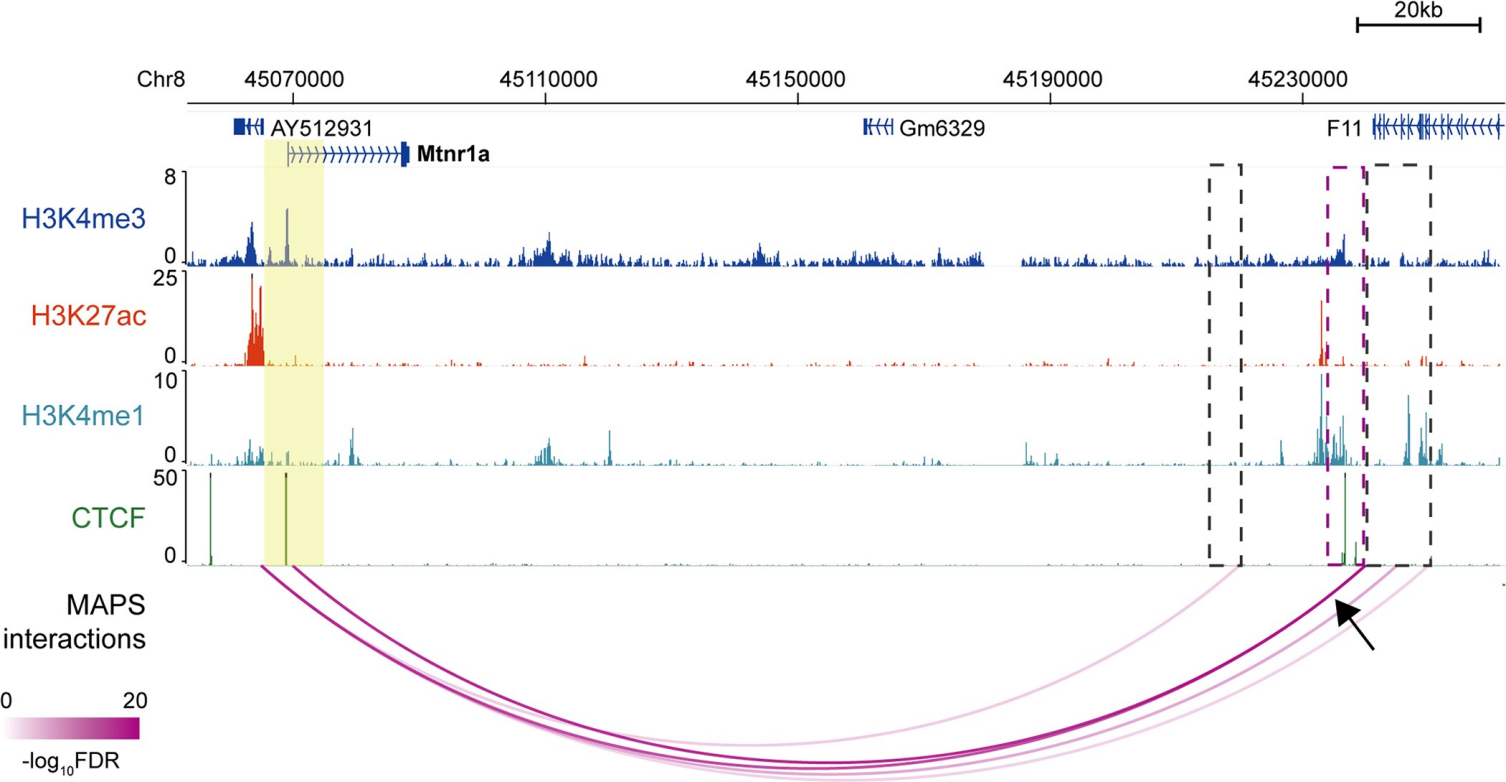
Genome-browser shows MAPS-identified interactions anchored at Mtnr1a promoter from mESC H3K4me3 PLAC-seq data. Anchor regions around Mtnr1a promoter are highlighted by yellow box (chr8:45,065,000–45,075,000, two 5Kb bins). The MAPS-identified interactions overlapping this anchor region are marked by magenta arcs. The black arrow points to the interaction verified in the previous publication [20] and the other end of the interaction is marked by magenta box. Additional interacting regions identified by MAPS are marked by grey boxes. No interaction is identified by hichipper anchored at this region from mESC H3K4me3 PLAC-seq data.

To further evaluate the performance of MAPS and hichipper at specific loci, we examined ten 3C/4C-verified long-range interactions centered at seven different promoters in mESCs from previous studies [16–20]. Among them, eight were recapitulated by MAPS using mESC H3K4me3 PLAC-seq data. By contrast, only five of them were found by hichipper using the same data (**S8 Table**). At these promoters MAPS also detected additional long-range interactions and most of the additional promoter-interacting regions are enriched in H3K4me1, CTCF or H3K27ac, suggesting that MAPS identified biologically relevant interactions (see more below) (**Fig 4** and **S5 Fig**).

### MAPS identifies biologically relevant interactions

**Fig 5:**
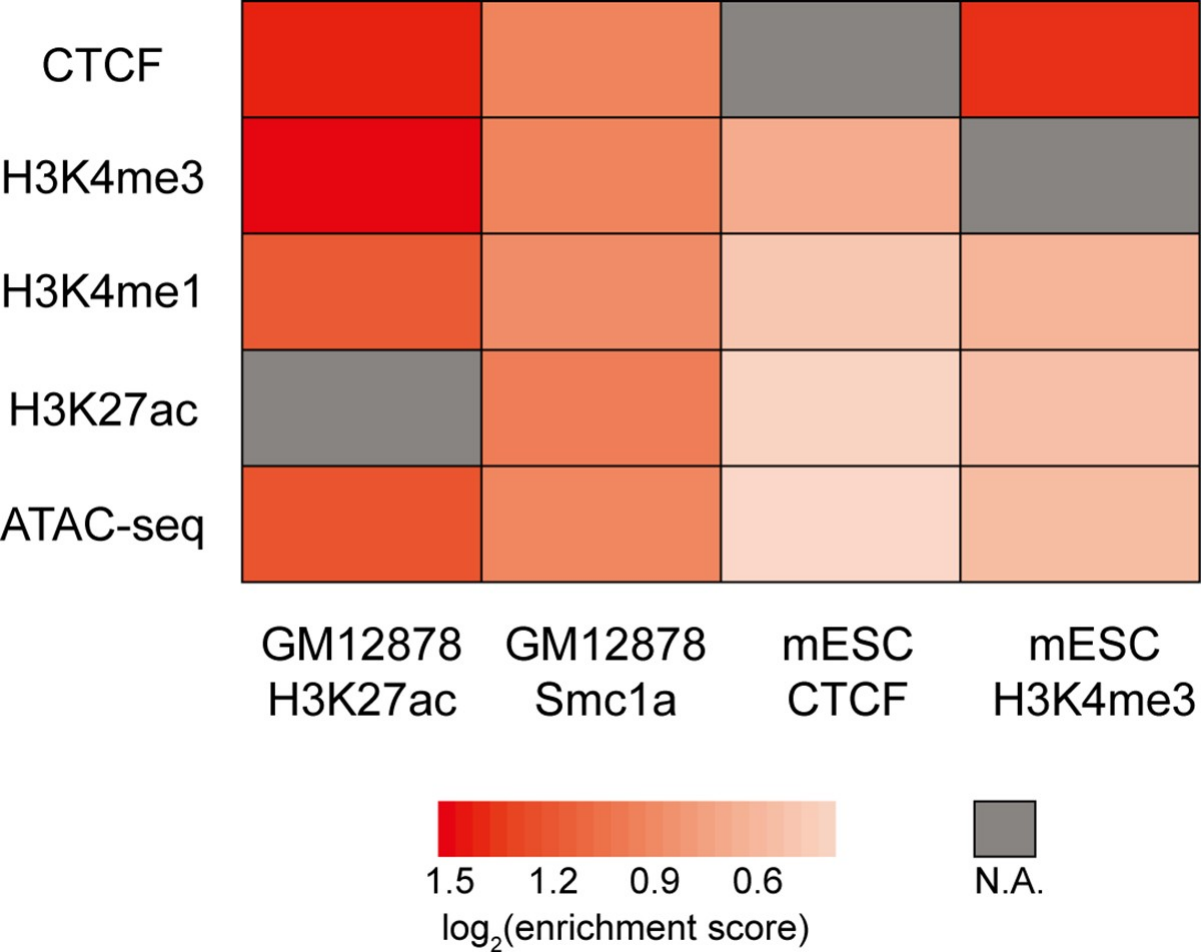
Cis-regulatory elements are enriched in the target bins of MAPS-identified “XOR” interactions. As only interactions from the “XOR” set are considered, CTCF enrichment analysis is not applicable for mESC CTCF PLAC-seq data, H3K4me3 enrichment analysis is not applicable for mESC H3K4me3 PLAC-seq data, and H3K27ac enrichment analysis is not applicable for GM12878 H3K27ac HiChIP data (denoted as N.A. in the heatmap). For each ChIP-seq/ATAC-seq data, we calculated the proportion of target bins and control bins containing ChIP-seq/ATAC-seq peaks, defined as %target and %control, respectively. We further defined the enrichment score as the ratio between %target and %control (numbers in **S9 Table**).

To thoroughly evaluate the biological relevance of MAPS-identified interactions, we first checked whether their anchor regions are enriched for *cis*-regulatory elements (CREs) that may contribute to gene regulation. Since MAPS-identified interactions always have at least one side of anchor overlapping the protein of interest and such protein-binding anchors may introduce bias to the enrichment analysis, we only selected the anchor bin that is not bound by the protein of interest from the “XOR” set of interactions (hereafter referred to as the “target” bin) for this analysis. Intersecting those target bins with H3K4me1, H3K4me3, H3K27ac, CTCF ChIP-seq and ATAC-seq peaks from matching cell types revealed that all these proteins are enriched 1.3 to 2.8 folds at target bins for all four PLAC-seq/HiChIP datasets (all Chi-square test p-values < 2.2e-16, **Fig 5**, **S6 Fig** and **S9 Table**).

Next we asked whether genes involved in MAPS-identified interactions tend to have higher expression level than those genes that are not. Previous studies demonstrated the positive correlation between transcriptional activity and the presence of promoter-centered long-range interactions [21, 22]. Indeed, the genes with their TSS overlapping with MAPS-identified interactions express 1.2 to 4.1 folds higher than the genes that are not overlapping (**S7 Fig** and **S10 Table**). Together the above results suggest that MAPS can call long-range interactions that are related to gene regulation.

### MAPS identifies numerous chromatin interactions missed by HiCCUPS from in situ Hi-C data

**Fig 6:**
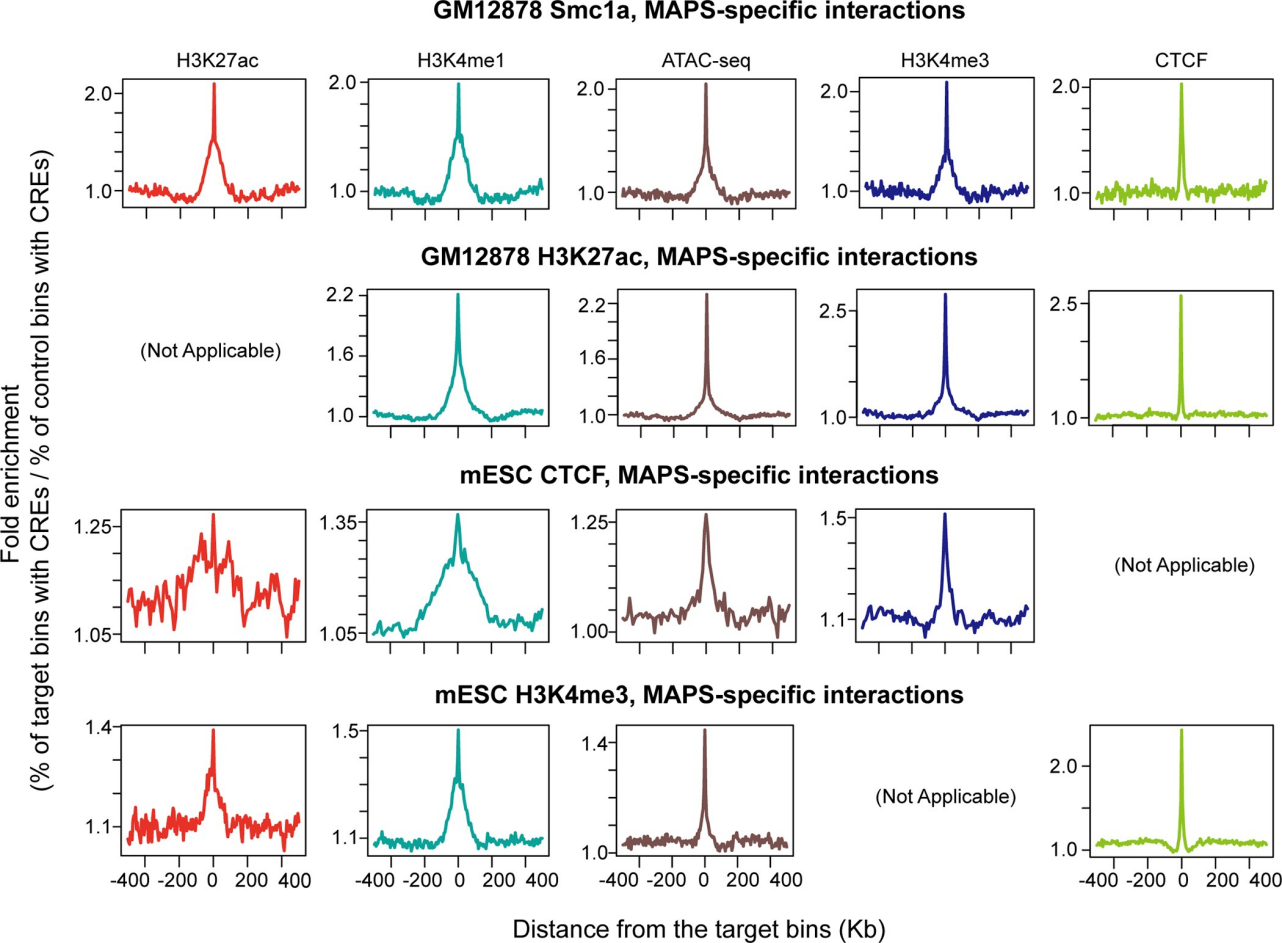
Enrichment of CREs around the target bins of XOR set of MAPS-specific interactions. Enrichment of H3K27ac (ChIP-seq peaks), H3K4me1 (ChIP-seq peaks), ATAC-seq peaks, H3K4me3 (ChIP-seq peaks) and CTCF (ChIP-seq peaks) in a window of 500Kb around the target bins for all four datasets. Due to the definition of XOR set of interactions, H3K27ac, H3K4me3 and CTCF enrichment level is not analyzed for GM12878 H3K27ac HiChIP, mESC H3K4me3 and mESC CTCF PLAC-seq data, respectively.

**Fig 7:**
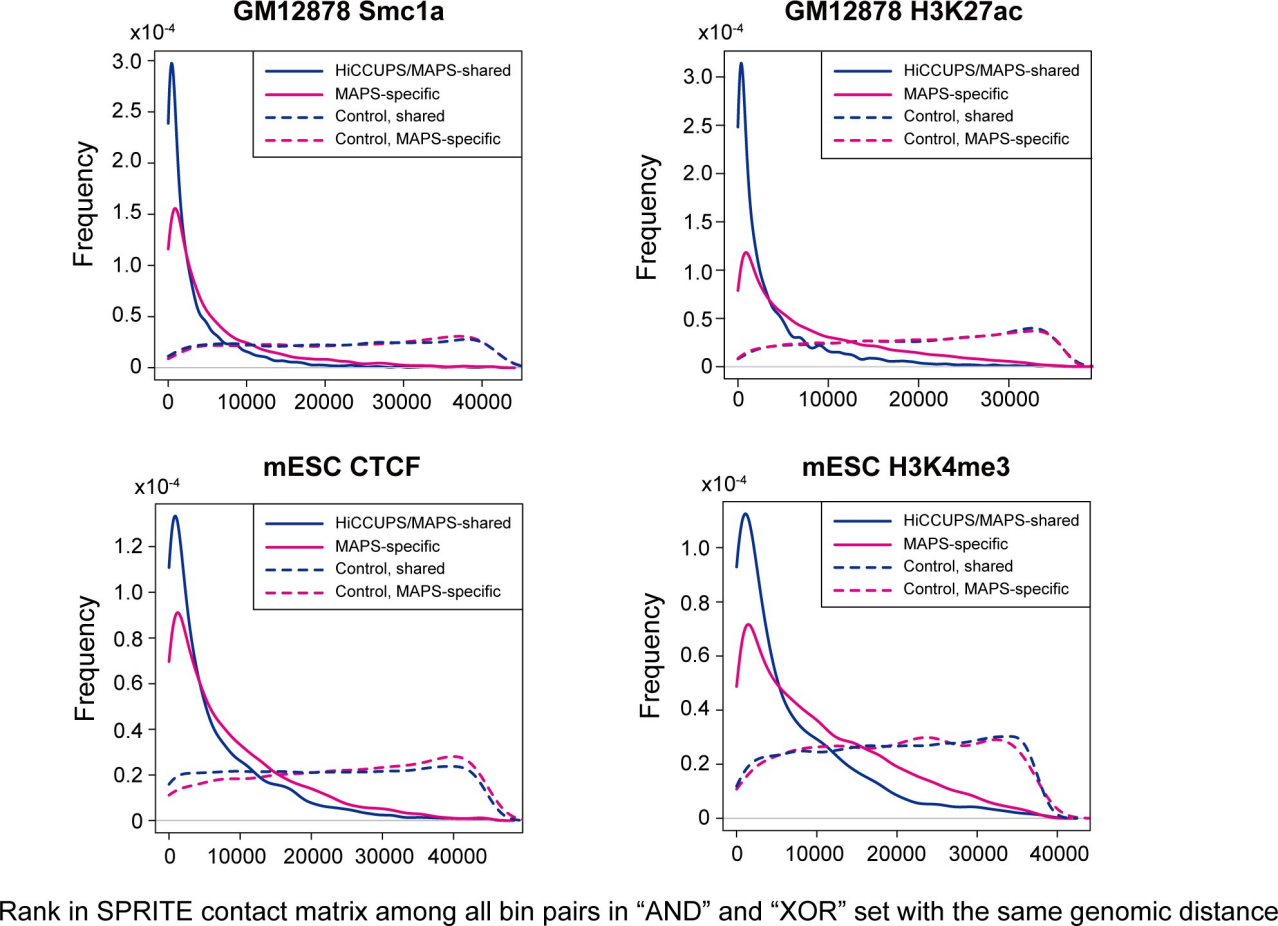
Frequency of MAPS-identified interactions and control bin pairs versus their rankings in the SPRITE contact matrix (see Methods for details). A bin pair with higher normalized SPRITE interaction frequency tends to rank top, among all bin pairs with the same genomic distance (only bin pairs in “AND” and “XOR” sets from the SPRITE contact matrix are considered).

**Fig 8:**
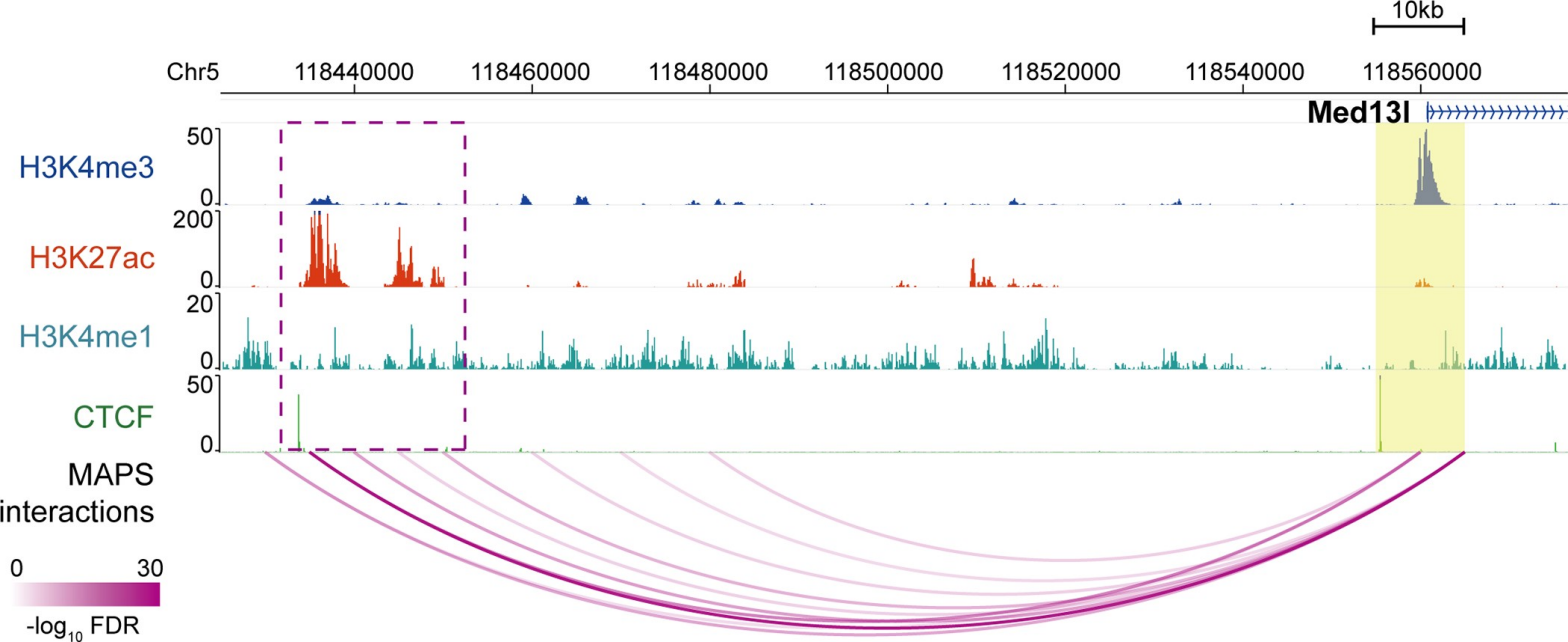
MAPS-identified interactions from mESC H3K4me3 PLAC-seq data anchored at Med13l promoter. Anchor region around target promoter is highlighted by yellow box. The MAPS-identified interactions overlapping this anchor region are marked by magenta arcs. The deleted enhancer region in Moorthy et al study [24] is marked by magenta box.

Since a large proportion (52.6% - 88.3%) of MAPS-identified interactions do not overlap with chromatin loops identified from in situ Hi-C data by HiCCUPS, we would like to further validate these MAPS-specific interactions (**S11 Table**). Several lines of evidence support the validity of these additional chromatin interactions. First, the “XOR” set of MAPS-specific interactions are enriched for H3K4me3, H3K4me1, H3K27ac, CTCF and ATAC-seq signal to the similar degree as the interactions called by both HiCCUPS (from in situ Hi-C data) and MAPS (from PLAC-seq or HiChIP datasets) (**Fig 6** and **S8 Fig**). Second, both HiCCUPS/MAPS-shared and MAPS-specific interactions show significantly higher contact frequency (all Wilcoxon test p-values < 2.2e-16) than the matched control set in the SPRITE data (split-pool recognition of interactions by tag extension) [23], an orthogonal method for mapping 3D chromatin structure independent of proximity ligation (**Fig 7**, see **Methods** for details). Third, the MAPS-identified enhancer-promoter interactions match better with functionally validated enhancer-promoter pairs compared to HiCCUPS-identified ones. A recent study revealed multiple such pairs in mESC via CRISPR/Cas9-mediated deletion of enhancers [24]. For the five promoter-enhancer pairs spanning a distance greater than 50Kb, MAPS is able to identify three of them (**Fig 8** and **S9 Fig**). By contrast, none of these five enhancer-promoter pairs are identified by HiCCUPS from in situ Hi-C data (**S12 Table**). All together these results indicate that MAPS can identify biologically relevant long-range chromatin interactions from PLAC-seq/HiChIP data with better sensitivity, compared to HiCCUPS-identified interactions from in situ Hi-C data.

## Discussion

The rapidly growing popularity of PLAC-seq and HiChIP technologies necessitates the development of effective data analysis method tailored to the new datasets. MAPS takes into account PLAC-seq/HiChIP-specific biases introduced by the ChIP procedure, and identifies long-range chromatin interactions anchored at different proteins with high reproducibility and accuracy. More importantly, MAPS can detect a large number of biologically relevant chromatin interactions that are missed by the state-of-the-art mapping approaches, making it a useful tool for investigators working on chromatin architecture, epigenomics, and gene regulatory networks.

Our current implementation of MAPS aims to identify intra-chromosomal long-range chromatin interactions at 5Kb or 10Kb bin resolution within 1Mb genomic distance, but it can be further extended. We found that MAPS also works well at finer resolution (2Kb bin resolution, **Note 4 in S1 Text**) or at extended genomic distance range (up to 2Mb, **Note 5 in S1 Text**) when the sequencing depth is sufficient. In addition, one can use the similar statistical framework to detect biologically relevant inter-chromosomal chromatin interactions [25]. Moreover, when the haplotype information is available, one can study allelic-specificity among MAPS-identified interactions. Last but not least, it is essential to apply MAPS to multiple PLAC-seq/HiChIP datasets and identify differential chromatin interactions which are specific to certain cell types or experimental conditions. These further developments of MAPS are beyond the scope of our current study, and we will pursue such directions in the near future.

We have released MAPS as a stand-alone software package with detailed user tutorial and sample input and output files. It can be freely downloaded from GitHub website: https://github.com/ijuric/MAPS. **S10 Fig** contains the MAPS running time for the PLAC-seq and HiChIP datasets used in this study. In general, MAPS running time increases linearly with the overall sequencing depth. The majority of computation time is at the MAPS pre-processing step.

In addition to the development of a novel software package MAPS, we have also provided two new PLAC-seq datasets (mESC CTCF PLAC-seq and mESC H3K4me3 PLAC-seq), which have been deposited to GEO with access number GSE119663. Noticeably, our mESC H3K4me3 PLAC-seq data is deeply sequenced, containing >1.1 billion raw reads (combining two biological replicates together). These data not only provide a highly valuable resource to study high resolution long-range chromatin interactions in mESCs, but also can be used to benchmark additional methods designed for PLAC-seq/HiChIP data analysis.

## Methods

### Sequencing data

All data sets used (both external and in-house generated for this study) are summarized in **S13 Table**.

### MAPS- and hichipper-identified interactions

The full list of MAPS- and hichipper-identified interactions (both default output and converted/filtered output) for all four datasets are provided in **S1 Data**. The format of files with extension “bedpe” is as below: fields 1–3 represents the genome coordinates for the left bins of interactions; fields 4–6 represents the genome coordinate for the right bins of interactions; field 7 represents the observed number of raw counts supporting this interaction; fields 8 represents the expected number of counts between the two bins calculated from MAPS (this field is always 0 for hichipper since hichipper does not calculate expected value); fields 9 is the FDR of the interaction calculated from MAPS/hichipper; field 10 is the interaction type (either as singletons or as part of a cluster); field 11 tells whether this interaction is the cluster summit if its interaction type is “cluster” (1 as yes and 0 as no; field 11 is 0 for all singletons). The files with extension “mango” are the default output of hichipper.

### Cell culture and fixation

The F1 Mus musculus castaneus × S129/SvJae mouse ESC line (F123 line) was a gift from Dr. Rudolf Jaenisch and was previously described [26]. F123 cells were cultured in DMEM (10013-CV, Corning), supplement with 15% knockout serum replacement (10828028, Invitrogen), 1×penicillin/streptomycin (15140122, Thermo Fisher Scientific), 1×non-essential amino acids (11140050, Thermo Fisher Scientific), 1×GlutaMax (35050061, Thermo Fisher Scientific), 1000 U/ml LIF (ESG1107, Millipore), 0.1 mM β-mercaptoethanol (M3128, Sigma). F123 cells were maintained on irradiated CF1 mouse embryonic fibroblasts (A34180, Thermo Fisher Scientific) and were passaged once on 0.1% gelatin-coated feeder-free plates before harvesting.

Cells were harvested by accutase treatment and resuspended in culture medium described above but without knockout serum replacement at a concentration of 1x10^6^ cells per 1ml. Methanol-free formaldehyde solution was added to a final concentration of 1% (v/v) and fixation was performed at room temperature for 15 min with slow rotation. The fixation was quenched by addition of 2.5 M glycine solution to a final concentration of 0.2 M with slow rotation at room temperature for 5 min. Fixed cells were pelleted by centrifugation at 2,500×*g* for 5 min at 4°C and washed with ice-cold PBS once. The washed cells were pelleted again by centrifugation, snap-frozen in liquid nitrogen and stored at -80°C.

### PLAC-seq on F123 cells

PLAC-seq libraries were prepared using method as previously described [5]. The detailed experimental procedures are provided in **Note 9 in S1 Text**. In brief, 1–3 million crosslinked F123 cells were digested 2 hours at 37°C using 100 U MboI followed by biotin fill-in and proximity ligation at room temperature for 4 hours. Then the nuclei were further lysed, sonicated and immunoprecipitated against the antibodies of choice. After immunoprecipitation, reverse crosslink was performed overnight at 65°C after adding proteinase K to extract DNA. DNA fragments containing ligation junctions were enriched with streptavidin beads followed by on-beads end repair, A-tail adding, adapter ligation and PCR amplification for 12–13 cycles.

### ATAC-seq on F123 cells

ATAC-seq was performed using method as previously described [27]. In brief, 100,000 freshly harvested F123 cells were resuspend in lysis buffer (10 mM Tris-HCl, pH 7.4, 10 mM NaCl, 3 mM MgCl_2_ and 0.1% IGEPAL CA-630) and rotate at 4°C for 15 minutes. After lysis the nuclei was spun down at 500×*g* for 5 min at 4°C. Then the reaction was carried out for 30 min at 37°C in 1×TD buffer with 2.5 μL transposase from Nextera DNA Library Prep Kit (Illumina). After reaction completion DNA is purified using MinElute PCR Purification Kit (Qiagen). PCR amplification was performed with 1×NEBNext PCR MasterMix and 1 μM i7-index and i5-index primers using the following PCR condition: 72°C for 5 min; 98°C for 30 s; and 8 cycles of 98°C for 10 s, 63°C for 30 s and 72°C for 1 min. The amplified libraries are purified and size selected using 0.55× and 1.5× (total) of sample volume.

### ChIP-seq on F123 cells

2 million fixed F123 cells were thawed on ice, resuspend in hypotonic lysis buffer (20 mM HEPES, pH 8.0, 10 mM KCl, 1 mM EDTA, 10% glycerol) with proteinase inhibitors and rotate at 4°C for 15 minutes. The nuclei were then washed once with hypotonic lysis buffer with proteinase inhibitors and resuspend in 130 μL RIPA buffer (10 mM Tris, pH 8.0, 140 mM NaCl, 1 mM EDTA, 1% Triton X-100, 0.1% SDS, 0.1% sodium deoxycholate) with proteinase inhibitors. After incubation on ice for 10 minutes, the nuclei were sheared using Covaris M220 with following setting: power, 75 W; duty factor, 10%; cycle per burst, 200; time, 10 minutes; temp, 7°C. The cell lysate was cleared by centrifugation at 15,000×*g* for 20 min and supernatant was collected. The clear cell lysate was precleared with Protein G Sepharose beads (GE Healthcare) and for 3 hours at 4°C with slow rotation. ~5% of precleared cell lysate was saved as input control. The rest of the lysate was mixed with 2.5 μg of H3K4me3 (04–745, Millipore) antibody and rotate at 4°C for at least 12 hours. On the next day, 0.5% BSA-blocked Protein G Sepharose beads (prepared one day ahead) were added and rotated for another 3 hours at 4°C. The beads were collected by centrifugation at 400×*g* for 1 min and then washed with RIPA buffer three times, high-salt RIPA buffer (10 mM Tris, pH 8.0, 300 mM NaCl, 1 mM EDTA, 1% Triton X-100, 0.1% SDS, 0.1% sodium deoxycholate) twice, LiCl buffer (10 mM Tris, pH 8.0, 250 mM LiCl, 1 mM EDTA, 0.5% IGEPAL CA-630, 0.1% sodium deoxycholate) once, TE buffer (10 mM Tris, pH 8.0, 0.1 mM EDTA) twice. Washed beads were treated with 10 μg Rnase A in extraction buffer (10 mM Tris, pH 8.0, 350 mM NaCl, 0.1 mM EDTA, 1% SDS) for 1 hours at 37°C, followed by reverse crosslinking in the presence of proteinase K (20 μg) overnight at 65°C. After reverse crosslink the DNA was purified by Zymo DNA Clean&Concentrator. For library preparation, 10–100 ng ChIP DNA or input DNA was first end repaired at 20°C for 30 minutes in 1×T4 DNA ligase buffer (NEB) with 0.5mM dNTP mix, 3U T4 DNA polymerase (NEB), 2.5U Klenow fragment (NEB) and 10U T4 PNK (NEB). The repaired DNA was then purified by Zymo DNA Clean&Concentrator and adenylated at 37°C for 30 minutes in 1×NEBbuffer 2 (NEB) with 0.4mM dATP, 10U Klenow fragment (3’-5’ exo-) (NEB). The adenylated DNA was purified by Zymo DNA Clean&Concentrator and ligated to the adapters (Illumina, TruSeq, 0.1 μL per 100ng DNA) at 16°C for overnight in 1×T4 DNA ligase buffer (NEB) with 400U T4 DNA ligase. After purification with Zymo DNA Clean&Concentrator, DNA was amplified with KAPA HiFi HotStart ReadyMix PCR Kit for 12 cycles according to the manufacturer’s instructions. The amplified libraries were purified with Ampure Beads to extract fragments between 200-600bp for sequencing.

### ChIP-seq data processing

The H3K4me3 ChIP-seq data on F123 cells was analyzed using ENCODE Uniform processing pipeline for ChIP-seq (histone marks) (https://github.com/ENCODE-DCC/chip-seq-pipeline) with default parameters.

### ATAC-seq data processing

ATAC-seq reads were mapped to mm10 genome using bowtie 1.1.2 with flags "-X2000—no-mixed—no-discordant". The reads were converted to bam files, sorted, and PCR duplicates and mitochondrial reads were removed using samtools. To account for the Tn5 insertion position, read end positions were moved 4bp towards the center of the fragment. Bigwig signal tracks and peak calls were generated using MACS2 2.1.1.20160309 and the following flags: "-nomodel -shift 37 -ext 73 -pval 1e-2 -B -SPMR -call-summits". To obtain the set of replicated peaks for each sample, the data were processed as described above for each replicate independently as well as pooled. Using bedtools 2.27.1, the pooled peaks were intersected against each replicate's peaks sequentially, and pooled peaks present in both replicates were considered to be 'replicated'.

### MAPS pre-processing component

MAPS took the raw paired-end reads (fastq files) from PLAC-seq and HiChIP experiment as input, mapped them to the reference genome (**S1 Fig**). Specifically, we used “bwa mem” to map each end of paired-end reads to the reference genome separately (mm10 or hg19, **S2 Table**), and removed non-mappable reads and low mapping quality reads. We further removed read pairs with less than two or more than three alignments. The read pairs with only one alignment contain no information whereas the chance of a read pair spanning two real ligation junctions (having more than three alignments) are rare and such pairs most likely represent spurious ligation events. A read pair is defined as “valid” when it has exactly two alignments. For a read pair with three alignments, in theory it can generate three different alignment pairs and we only chose one “valid” alignment pair from each read pair to avoid counting the same ligation event multiple times. The choice of valid alignment pair is based on the following roles: 1) if all three alignments are on the same chromosome, it often suggests one of the reads spans the ligation junction. In this case, the alignment pair with the second largest linear distance is defined as “valid”, since it represents the pair that is closer to the ligation junction. If three alignments are on two different chromosomes, in most cases the two alignments within the same chromosome are close to each other, therefore we randomly selected one of the two alignments on the same chromosome, and pair with the alignment on the other chromosome. The chance of three alignments are on three different chromosomes is very low and such pairs most likely represent spurious ligation events (the chance of a read pair spanning two real inter-chromosomal ligation junctions is low), therefore we discard such pairs. After pairing all the reads as described above, we used “samtools rmdup” to remove PCR duplicates. Furthermore, we split the reads into two groups in to short-range reads (< = 1Kb) and long-range reads (>1Kb). The short-range reads (< = 1Kb) are used to correct the bias introduced by ChIP in subsequent normalization since they are more likely to be dangling ends or self-ligation products of undigested DNA rather than the products of proximity ligation. We chose 1Kb as the distance cutoff because the insert size of PLAC-seq/HiChIP libraries are often less than 1Kb. We checked the strand orientations of the two ends of short-range reads (< = 1Kb) in all four PLAC-seq/HiChIP datasets used in this study and found only 2–8% of them have two ends mapped to the same strand, suggesting that the percentage of proximity ligation artifacts in the short-range reads is low. We then further filtered these known proximity ligation artifacts from short-range reads to generate the final short.bed file. On the other hand, long-range reads (>1Kb) were used to identify long-range interactions. MAPS extracted the intra-chromosomal long-range reads and took the ChIP-seq peaks of protein of interest as the interaction anchors (**S13 Table**), and grouped all bin pairs into the “AND”, “XOR” and “NOT” sets. MAPS only selected the “AND” and “XOR” sets for the next data normalization step. Notably, the current version of MAPS requires a list of protein binding peaks as the input to determine the interaction anchors. When the ChIP-seq data is not available, one can apply hichipper to PLAC-seq and HiChIP data to obtain interaction anchors. Since hichipper has already achieved good performance for 1D anchor identification from PLAC-seq and HiChIP data, our MAPS method only focuses on the identification of statistically significant long-range chromatin interactions.

Data normalization is a challenging issue for any chromatin interaction data. Notably, the matrix-balancing algorithms used for Hi-C data normalization, including ICE [28], VC [29] and KR [4], are inappropriate for PLAC-seq and HiChIP data normalization. Due to the ChIP procedure, in theory the bins with protein binding always have much higher total number of contacts compared to the bins without protein binding, which violates the crucial “equal visibility” assumption implied by the matrix-balancing algorithms.

To accommodate unique features of PLAC-seq and HiChIP data, we propose to extend our previous HiCNorm [13] method to normalize PLAC-seq and HiChIP data. Let *x*_*ij*_ represent the read count (i.e., number of paired-end reads) spanning between bin *i* and bin *j*. Due to symmetry, we only considered bin pairs (*i*,*j*) with *i*<*j*. In addition, we only considered intra-chromosomal contacts within 1Mb, and did not use two adjacent bin pairs. Let *f*_*i*_, *gc*_*i*_, *m*_*i*_ and *IP*_*i*_ represent the effective fragment length, GC content, mappability score, and ChIP enrichment level (measured by the number of short-range reads, i.e., intra-chromosomal reads < = 1Kb) of bin *i*, respectively. The definition of *f*_*i*_, *gc*_*i*_ and *m*_*i*_ are described in HiCNorm [13]. Specifically, we first truncated each fragment end up to 500 bp, and then defined the effect fragment length of bin *i* (*f*_*i*_) as the total length of truncated fragment end within bin *i*. Next, we calculated GC content and mappability score for each fragment end, and then defined the GC content of bin *i* (*gc*_*i*_) and mappability score of bin *i* (*m*_*i*_) as the average GC content and mappability score of all fragment ends within bin *i*, respectively. *f*_*i*_, *gc*_*i*_ and *m*_*i*_ for human genome and mouse genome at different bin resolutions can be downloaded from the following website: http://enhancer.sdsc.edu/yunjiang/resources/genomic_features/. Since at kilobase resolution the PLAC-seq and HiChIP data are extremely sparse, and our goal is to identify statistically significant long-range chromatin interactions, we only modeled bin pairs (*i*,*j*) with non-zero count (*x*_*ij*_≥1), and assumed that *x*_*ij*_ follows a zero-truncated Poisson (ZTP) distribution with mean *μ*_*ij*_ (**Note 3 in S1 Text**), where

$$\log\left(\mu_{ij}\right)=\beta_{0}+\beta_{f}\;\log\left(f_{i}*f_{j}\right)+\beta_{GC}\;\log\left(gc_{i}*{gc}_{j}\right)+\beta_{m}\;\log\left(m_{i}*m_{j}\right)+\beta_{IP}\;\log\left({IP}_{i}*{IP}_{j}\right)+\beta_{d}\;\log\left(d_{ij}\right).$$

Here *β*_0_ is the intercept for overall sequencing depth. *β*_*f*_, *β*_*GC*_, *β*_*m*_, *β*_*IP*_ and *β*_*d*_ are regression coefficients for effective fragment length, GC content, mappability score, ChIP enrichment level and genomic distance, respectively. *d*_*ij*_ denotes the genomic distance between bin *i* and bin *j*. We fit the aforementioned ZTP regression model for each chromosome, separately for the “AND” set and the “XOR” set, using R function “ppois” in the “VGAM” library, to obtain the expected contact frequency *e*_*ij*_ for each bin pair (*i*,*j*). These *e*_*ij*_’s represent background expected from random chromatin collisions. Next, we calculated a ZTP p-value for each bin pair (*i*,*j*), defined as *p*_*ij*_ = *ZTP*(*X*>*x*_*ij*_|*e*_*ij*_). Similar to Fit-Hi-C, we viewed bin pairs with extremely low p-values (< 1 / total number of non-zero bin pairs) as outliers. We then removed those outliers, and re-fit the ZTP regression model using the remaining data to re-calibrate the background, obtaining re-calibrated expected contact frequency ${\tilde{e}}_{ij}$ and corresponding ZTP p-value ${\tilde{p}}_{ij}=ZTP(X>x_{ij}|{\tilde{e}}_{ij})$. We further converted ZTP p-value ${\tilde{p}}_{ij}$ into false discovery rate (FDR) *q*_*ij*_ using R function “p.adjust”. Within each chromosome, the FDR was calculated by the “AND” and “XOR” set, separately.

### MAPS interaction calling component

We then identified statistically significant long-range chromatin interactions from normalized PLAC-seq and HiChIP data. Specifically, we defined a bin pair (*i*,*j*) as a statistically significant bin pair if it satisfies the following three criteria simultaneously: (1) *x*_*ij*_≥12, (2) $x_{ij}/{\tilde{e}}_{ij}\geq 2$ and (3) *q*_*ij*_<0.01. Details of justification of such threshold values can be found in **Note 6 in S1 Text**. Notably, the threshold values used in MAPS may be defined by users depending on the sequencing depth of available PLAC-seq/HiChIP data and the purpose of analysis. Since the sequencing depth of all four datasets used in this study is relatively high, we chose such stringent threshold to minimize the potential false positive calls. Starting from these significant bin pairs, we further grouped adjacent ones into clusters, and singletons (defined as isolated significant bin pairs without adjacent ones). Specifically, we denoted *d*_*ij*_ as the genomic distance between bin *i* and bin *j*, and grouped significant bin pair (*i*,*j*) and significant bin pair (*m*,*n*) into the same interaction cluster if max{*d*_*im*_,*d*_*jn*_}≤15Kb. Each significant bin pair belongs to one unique cluster, or it is a singleton. For the significant bin pairs defined as singletons, we applied additional filtering and only kept the ones with *q*_*ij*_<10^−4^ as significant interactions since singletons are more likely to be false positives. For the significant bin pairs as part of a cluster, we keep all of them as significant interactions [4]. For each interaction cluster, we further identified its summit, defined as the bin pair(s) with the lowest FDR. Therefore, the final MAPS output contains the following information: 1) a list of statistically significant long-range chromatin interactions; 2) for each interaction, whether it is a singleton or belongs to a cluster; 3) if an interaction is part of a cluster, whether it is the summit of this cluster and which interactions are in the same cluster. We repeated sensitivity and CTCF motif orientation analysis using only the sum of singletons and cluster summits and obtained consistent results (**S14 Table**), showing that MAPS performs equally well when restricted to a conservative subset of interaction calls.

To verify the robustness of MAPS, we further checked the overlaps between MAPS-identified interactions from mESC CTCF PLAC-seq data and those from mESC H3K4me3 PLAC-seq data. Our hypothesis is that the “real” interactions must be detectable from different PLAC-seq experiments in the same cell type even when different antibodies are used. Since PLAC-seq can only detect interactions with at least one end binding to the protein of interest, we only compared the MAPS-identified interactions from those two datasets on the “common” anchor regions with both H3K4me3 and CTCF binding. Specifically, we first defined “common” anchor bins as the ones containing both CTCF and H3K4me3 ChIP-seq peaks (**S15 Table**). The bin resolution is 10Kb and 5Kb for mESC CTCF PLAC-seq data and mESC H3K4me3 PLAC-seq data, respectively. Next, we selected the testable MAPS-identified interactions from CTCF (32,474 out of 53,788) and H3K4me3 PLAC-seq data (79,727 out of 134,179) with at least one end being the “common” anchor bin for comparison. We denoted *d*_*ij*_ as the genomic distance between bin *i* and bin *j*. We then defined an interaction, bin pair (*i*,*j*), overlaps with another interaction, bin pair (*m*,*n*), when max{*d*_*im*_,*d*_*jn*_}≤ 15Kb. With this definition, 90.3% testable interactions from mESC CTCF PLAC-seq data overlap with 69.1% testable interactions from mESC H3K4me3 PLAC-seq data, indicating that MAPS-identified interactions from the same cell type are highly consistent even when different proteins are targeted.

### Identification of interactions with hichipper

To call interactions from the same PLAC-seq and HiChIP datasets using hichipper, we performed the mapping and preprocessing using the default settings of HiC-Pro 2.7.6 and bowtie 2.3.0 as base mapper (recommended by hichipper), specifying digestion fragment size of 100 to 100,000. Genome fragment size files were obtained from the GitHub repository of hichipper (https://github.com/aryeelab/hichipper). Since the data in each run are from one sample and no merging was required, we removed allValidPairs and mRStat files to make the HiC-Pro output consistent with the requirements of the hichipper input. We then used hichipper v0.4.4 to call interactions using ChIP-seq peaks as interaction anchors (**S13 Table**). Notably, the default hichipper output is at interacting anchor resolution, which has a median size ~4Kb, and is not in the unit of 5Kb bin or 10Kb bin (files with extension “mango” in **S1 Data**). To make a fair comparison between MAPS and hichipper, we used the same threshold values described above for MAPS calls to filter the outputs from hichipper and then converted hichipper-identified interactions into 5Kb or 10Kb bin pairs. Specifically, we first filtered the hichipper output and only kept the hichipper interactions in autosomal chromosomes with raw contact frequency > = 12 and FDR < 1% (hichipper does not calculate the expected contact frequency, so the filter based on normalized contact frequency cannot be applied). Next, we partitioned each of these hichipper interactions into equal sized bin pairs (5Kb or 10Kb, depending on the resolution used in MAPS on the same dataset). Since a significant proportion of hichipper anchors are larger than 5Kb or 10Kb, one hichipper-identified interactions may be partitioned into multiple 5Kb or 10Kb interactions after this conversion. We then removed the 5Kb or 10Kb interactions falling into the XOR and NOT sets, and only kept those in the AND set after partition to: 1) avoid counting the same hichipper interaction multiple times; 2) make the converted hichipper interaction list having the same property as its default output (all anchor regions in default hichipper output contain at least one 1D ChIP-Seq peak). Afterwards we removed the interactions between two adjacent bins or with a genomic distance over 1Mb. We then grouped the remaining interactions into clusters or singletons using the same definition described above and kept all interaction clusters, and the singletons with a more stringent FDR < 0.0001 as the final hichipper-identified interaction list. The final converted hichipper output has the same format as the MAPS output.

### HiCCUPS loops from in situ Hi-C data

The HiCCUPS loops of GM12878 are acquired from Rao et al. study [4]. Specifically, file “GSE63525_GM12878_primary+replicate_HiCCUPS_looplist.txt.gz” was downloaded, which contains in total 9,448 loops. Among these 9,448 loops, we selected 6,316 loops where both two interacting anchors are 5Kb bins (**S5 Table**). To generate the 5Kb and 10Kb resolution of HiCCUPS loops of mESCs, we downloaded the raw fastq files of all four biological replicates from Bonev et al. study [14] and performed mapping, pairing reads and PCR duplicates removal in the same way as we did for PLAC-seq and HiChIP data (refer to “**MAPS pre-processing component”** above). Afterwards we combined the valid pairs from all four replicates and then applied HiCCUPS to call loops at 5Kb and 10Kb resolution with the following parameters: “-r 5000,10000 -k KR -f .1,.1 -p 4,2 -i 7,5 -t 0.02,1.5,1.75,2 -d 20000,20000” (**S5 Table**).

### Reproducibility analysis

We evaluated the reproducibility of MAPS- and hichipper-identified interactions between two biological replicates. We denoted *d*_*ij*_ as the genomic distance between bin *i* and bin *j*. We then defined an interaction, bin pair (*i*,*j*), in one replicate is reproducible, if and only if there exists an interaction, bin pair (*m*,*n*), in the other replicate such that max{*d*_*im*_,*d*_*jn*_}≤ 15Kb.

### Sensitivity analysis

We evaluated the sensitivity of MAPS- and hichipper-identified interactions, using HiCCUPS loops called from deeply sequenced in situ Hi-C datasets as true positives. Specifically, we used GM12878 in situ Hi-C data with ~4.9 billion reads from Rao et al. study [4], and mESC in situ Hi-C data with ~7.3 billion reads from Bonev et al. study [14]. We first selected a subset of HiCCUPS loops which are detectable in corresponding PLAC-seq and HiChIP data (**S6 Table** and **Note 8 in S1 Text**). Next, we defined a HiCCUPS loops, bin pair (*i*,*j*), is re-discovered by MAPS or hichipper, if and only if there exists an interaction, bin pair (*m*,*n*), called by MAPS or hichipper such that max{*d*_*im*_,*d*_*jn*_}≤ 15Kb. The sensitivity is calculated by the ratio between the number of HiCCUPS loops re-discovered by MAPS or hichipper and the total number of HiCCUPS loops detectable in PLAC-seq and HiChIP data.

Noticeably, although hichipper did not detect any loop in the “XOR” set (**S4 Table**), there are some testable HiCCUPS loops in XOR set recovered by hichipper (**S7 Table**). The reason is that our definition of loop overlap described above allows 15Kb gap and when the “XOR” set of HiCCUPS loops are close enough to the hichipper calls (which are always from the “AND” set) they are counted as “recovered” loops by hichipper.

### CTCF motif orientation analysis

We examined the CTCF motif orientation of testable MAPS- and hichipper-identified interactions. Specifically, we first download the CTCF ChIP-seq peak lists of GM12878 and mESC (**S13 Table**) and then searched for all the CTCF sequence motifs among those peak using FIMO [30] (default parameters) and the CTCF motif (MA0139.1) from the JASPAR [31] database. Based on this list of CTCF motifs, we then selected a subset of MAPS- or hichipper-identified interactions with both ends containing either single CTCF motif or multiple CTCF motifs in the same direction. Finally, we counted the frequency of four possible directionality of CTCF motif pairs, and calculated the proportion of convergent, tandem and divergent CTCF motif pairs among all testable interactions.

### *Cis*-regulatory elements enrichment analysis

For two interacting bins in the “XOR” set, we defined the bin which is bound by the protein of interest as the “anchor” bin, and the bin which is not bound by the protein of interest as the “target” bin. In order to access the biological relevance of peaks in the “XOR” set, we evaluated whether *cis*-regulatory elements are enriched within those target bins, compared to the control bins which are in the same distance with the anchor bin, but is not bound by the protein of interest (**S6 Fig**).

The ChIP-seq and ATAC-seq data used for this analysis is summarized in **S13 Table**. For each ChIP-seq/ATAC-seq data, we calculated the proportion of target bins and controls containing ChIP-seq/ATAC-seq peaks, defined as %target and %control, respectively. We further defined the enrichment score as the ratio between %target and %control.

### Definition of MAPS-specific interactions and HiCCUPS/MAPS-shared interactions (for Fig 6)

We divided all MAPS-identified interactions into two groups based on their overlap with HiCCUPS loops. Similar to our method in the sensitivity analysis, we defined a MAPS interaction, bin pair (*i*,*j*), is overlapped with a HiCCUPS loop, if and only if there exists an interaction, bin pair (*m*,*n*), in HiCCUPS loop list such that max{*d*_*im*_,*d*_*jn*_}≤ 15Kb. If a bin pair (*i*,*j*) is overlapped with a HiCCUPS loop, we defined it as a HiCCUPS/MAPS-shared interaction. If a bin pair (*i*,*j*) is not overlapped with a HiCCUPS loop, we defined it as a MAPS-specific interaction. The number of MAPS-specific interactions and HiCCUPS/MAPS-shared interactions are listed in **S11 Table**.

### Validation of MAPS-specific interactions by SPRITE data

The normalized SPRITE interaction frequency matrices were downloaded from GEO with access number GSE114242 [23]. The GM12878 and mESC SPRITE data is at 25Kb bin and 20Kb bin resolution, with reference genome hg19 and mm9, respectively. Since SPRITE matrix is at a lower resolution compared to our MAPS calls (5Kb or 10Kb), for this analysis we defined “MAPS-specific” and “HiCCUPS/MAPS-shared interactions” differently from what we described above and only used the singletons and the summits of interaction clusters from “MAPS-specific” or “shared” group for plot (related to **Fig 7**). Specifically, the MAPS-identified interactions consist of two types of bin pairs: singletons and interaction clusters (defined in “**MAPS interaction calling component**” section). For each singleton bin pair (*i*,*j*), we defined it as “shared” if there exists a bin pair (*m*,*n*) in HiCCUPS loop such that max{*d*_*im*_,*d*_*jn*_}≤ 15Kb. Otherwise, we defined the singleton bin pair (*i*,*j*) as “MAPS-specific”. For each interaction cluster, we defined it as “shared” if any one bin pair in the interaction cluster is a “shared” bin pair. Otherwise, if all bin pairs in an interaction cluster are “MAPS-specific”, we defined the entire interaction cluster as “MAPS-specific”. We then selected singletons and the summits of interaction clusters from “MAPS-specific” or “shared” group for the downstream analysis. We then zoomed the selected bin pairs out to the matched lower resolution in SPRITE data for a fair comparison. Specifically, for MAPS-identified interactions from GM12878 Smc1a and GM12878 H3K27ac HiChIP data, we first selected the center position of 5Kb interacting bin of an interaction summit, and then allocated the 25Kb bin containing that center position. This procedure created a list of 25Kb bin pair, among which each contains MAPS-identified interaction summit. Similarly, for MAPS-identified interactions from mESC CTCF and mESC H3K4me3 PLAC-seq data, we first selected the center position of 10Kb/5Kb interacting bin of an interaction summit (reference genome mm10), converted it into reference genome mm9 using UCSC Liftover tool (https://genome.ucsc.edu/cgi-bin/hgLiftOver), and then allocated the 20Kb bin containing that center position. This procedure created a list of 20Kb bin pair, among which each contains MAPS-identified interaction summit.

To evaluate the normalized SPRITE interaction frequency for MAPS-identified interactions, we used the following procedure to create the control set. For a bin pair in the “XOR” set, we defined the bin with ChIP-seq peak as the “anchor” bin and the bin without ChIP-seq peak as the “target” bin. We then find the “control” bin and such as the “anchor” bin has the same genomic distance between the “target” bin and the “control” bin (**S6 Fig**). The control bin pair is defined as the pair of the “anchor” bin and the “control” bin. For a bin pair in the “AND” set, since both two bins contain ChIP-seq peak, we randomly selected one bin as the “anchor” bin, and defined the remaining one as the “target” bin. Next, we repeated the procedures described above to find the “control” bin, and created the control bin pair for the bin pairs in the “AND” set. Finally, we filtered out any control bin pairs which are overlapped with MAPS-identified interactions. Let *S*_*ij*_ represent the normalized SPRITE interaction frequency between 25Kb/20Kb bin *i* and *j*. We defined the rank of bin pair (*i*,*j*) as the number of bin pair in the same genomic distance, but with higher normalized SPRITE interaction frequency than *S*_*ij*_. Here in the normalized SPRITE interaction frequency matrices, we only used all bin pairs in the “AND” and “XOR” set.

$$Rank(i,j)=\#\{S_{m,n}>S_{ij}:|m-n|=|i-j|,at\;least\;one\;of\;two\;bins\;(m,n)\;contains\;ChIP-seq\;peak.\}$$

A bin pair with higher normalized SPRITE interaction frequency tends to rank top, among all bin pairs with the same genomic distance.

### Gene expression analysis

To examine the correlation between MAPS-identified interactions and gene expression level, we first checked how many MAPS-identified interactions are overlapped with gene’s TSS. We then collected published RNA-seq data in mESCs and GM12878 cells [16, 32], and calculated fragments per kilobase of transcript per million mapped reads (FPKM) for each protein-coding gene. Next, we divided all protein-coding genes into two groups, based on whether their transcript start site (TSS) overlap MAPS-identified interactions and calculated their expression level.

## Supporting information

S1 Fig Flowchart of MAPS pre-processing steps. Details can be found in **Methods** section.
(TIF)

S2 Fig Contact frequency plots of chromosome 1 for all four datasets. The results are based on combined datasets after merging two biological replicates; 10Kb resolution is used for mESC CTCF and 5Kb resolution is used for all the other datasets. The X-axis is Log_10_ genomic distance between two interacting bins (unit: Kb). The Y-axis is the Log_10_ average raw PLAC-seq/HiChIP contact frequency. The red line, blue and purple lines represent the contact probability for bin pairs in the “AND”, “XOR” and “NOT” set, respectively.
(TIF)

S3 Fig Data normalization by MAPS. **(a) MAPS removes biases in GM12878 Smc1a HiChIP data after normalization.** For all autosomal chromosomes, we calculated the Pearson correlation coefficients (Y-axis) between the systemic biases (effective length, GC content, mappability, IP effect) and the raw contact frequency in the “AND” set, the normalized contact frequency in the “AND” set, the raw contact frequency in the “XOR” set and the normalized contact frequency in the “XOR” set, highlighted in red, yellow, blue and purple boxes, respectively. The grey dash line presents the Pearson correlation coefficient zero. Three panels show the results in replicate 1, replicate 2, and the combined data (replicate 1 + replicate 2), respectively. **(b-d)** Similar to **S3 Fig a**, **MAPS removes biases in GM12878 H3K27ac HiChIP data (b), mESC CTCF PLAC-seq data (c) and mESC H3K4me3 PLAC-seq data (d).**
(TIF)

S4 Fig Summary of MAPS- and hichipper-identified interactions of all four datasets. **(a) The number of interactions and the distribution of interaction length of MAPS-identified interactions.** From left to right are the results of MAPS calls from mESC CTCF PLAC-seq, mESC H3K4me3 PLAC-seq, GM12878 Smc1a HiChIP and GM12878 H3K27ac HiChIP combined data (replicate 1 + replicate 2). Each histogram shows the distribution of interaction length. The vertical blue bar represents the median distance of interactions. **(b)** Similar to **S4 Fig a**, **the number of interactions and the distribution of interaction length of hichipper-identified interactions.**
(TIF)

S5 Fig **MAPS-identified interactions from mESC H3K4me3 PLAC-seq data anchored at: (a) Pou5f1 promoter, (b) Sox2 promoter, (c) Tbx5 promoter, (d) Wnt6 promoter, (e) Nanog promoter.** Anchor regions around target promoter are highlighted by yellow boxes. The MAPS-identified interactions overlapping the anchor regions are marked by magenta arcs. The black arrow points to the interaction verified in previous publications [16–20] and the other end of the interaction is marked by magenta boxes. Additional interacting regions identified by MAPS are marked by grey boxes.
(TIF)

S6 Fig Cartoon illustration of anchor bin, target bin and control bin used in *cis*-regulatory elements enrichment analysis (related to Fig 5, Fig 6, S8 Fig and S9 Table). The solid yellow curve at left represents a statistically significant long-range chromatin interaction in the “XOR” set, connecting the anchor bin (the red box) and the target bin (the blue box). The dashed yellow curve at right represents a random collision between the anchor bin (the red box) and the control bin (the purple box). The interaction and the random collision has the same genomic distance.
(TIF)

S7 Fig Gene expression data analysis. In each panel, the y-axis represents the log_2_(FPKM+1). The red box and blue box represent the genes in which TSSs are associated with MAPS-identified interactions and genes in which TSSs are not associated with MAPS-identified interactions, respectively. For all four datasets, genes in which TSS involves with MAPS-identified interactions have significantly higher expression than genes in which TSS does not involve with MAPS-identified interactions (p<2.2e-16).
(TIF)

S8 Fig Enrichment of CREs around the target bins of XOR set of HiCCUPS/MAPS-shared interactions. Enrichment of H3K27ac (ChIP-seq peaks), H3K4me1 (ChIP-seq peaks), ATAC-seq peaks, H3K4me3 (ChIP-seq peaks) and CTCF (ChIP-seq peaks) in a window of 500Kb around the target bins for all four datasets. Due to the definition of XOR set of interactions, H3K27ac, H3K4me3 and CTCF enrichment level is not analyzed for GM12878 H3K27ac HiChIP, mESC H3K4me3 and mESC CTCF PLAC-seq data, respectively.
(TIF)

S9 Fig **MAPS-identified interactions from mESC H3K4me3 PLAC-seq data anchored at: (a) Elt4 promoter (chr2:20,515,000–20,525,000), (b) Ifitm3 promoter (chr7:141,005,000–141,015,000).** Anchor regions around target promoters are highlighted by yellow boxes. The MAPS-identified interactions overlapping this anchor region are marked by magenta arcs. The deleted enhancer regions in Moorthy et al study [24] are marked by magenta boxes.
(TIF)

S10 Fig MAPS running time (Y-axis) increases linearly proportional to overall sequencing depth (X-axis). (TIF)

S1 Table Summary statistics of AND, XOR and NOT set of bin pairs on chromosome 1. (XLSX)

S2 Table A summary of four PLAC-seq and HiChIP datasets used in this study. (XLSX)

S3 Table Reproducibility of MAPS and hichipper between two biological replicates. (XLSX)

S4 Table Summary of MAPS- and hichipper-identified interactions from combined datasets. (XLSX)

S5 Table A brief summary of HiCCUPS loops identified from deeply sequenced in situ Hi-C data. (XLSX)

S6 Table Number of HiCCUPS loops which are detectable in each PLAC-seq and HiChIP data. (XLSX)

S7 Table Overlap between testable HiCCUPS loops and MAPS-identified or hichipper-identified interactions. (XLSX)

S8 Table Ten long-range promoter-centered interactions in mESC verified by 3C or 4C in previous publications [16–20]. (XLSX)

S9 Table Numbers related to *cis*-regulatory elements enrichment analysis. (XLSX)

S10 Table Numbers MAPS-identified interactions overlapping with gene TSS. (XLSX)

S11 Table Overlap between MAPS-identified interactions and HiCCUPS loops in all four PLAC-seq and HiChIP datasets. (XLSX)

S12 Table A list of functionally validated enhancer-promoter pairs in mESC from Moorthy et al study [24]. Only enhancer-promoter pairs are >50Kb in genomic distance are listed.
(XLSX)

S13 Table Summary of all sequencing data sets used in this study. (XLSX)

S14 Table Results of sensitivity and CTCF motif orientation analysis from only cluster summits and singletons. (XLSX)

S15 Table Number of common anchor bins containing both CTCF and H3K4me3 ChIP-seq peaks in mESC. (XLSX)

S1 Text Supporting information. **Note 1**. Similarities and differences between PLAC-seq and HiChIP protocols. **Note 2.** Fit-Hi-C, HiCCUPS, Mango and hichipper are not optimal for the identification of long-range chromatin interactions from PLAC-seq and HiChIP data. **Note 3.** Justification of zero-truncated Poisson model used in MAPS. **Note 4.** MAPS analysis at finer resolution. **Note 5.** MAPS analysis with extended genomic distance range. **Note 6.** Justification of threshold values used in the MAPS interaction calling component. **Note 7.** Reproducibility of HiCCUPS loops. **Note 8.** Selecting HiCCUPS loops which are detectable in PLAC-seq and HiChIP data. **Note 9.** Detailed experimental procedures of PLAC-seq on F123 cells.
(DOCX)

S1 Data The full list of MAPS- and hichipper-identified interactions (both default output and converted/filtered output) for all four datasets used in this study. (ZIP)
