# Supplementary figures and images for: MAPS: Model-based analysis of long-range chromatin interactions from PLAC-seq and HiChIP experiments
Source: PLoS Comput Biol. 2019 Apr 15;15(4):e1006982. doi: 10.1371/journal.pcbi.1006982 (PMC6483256; doi:10.1371/journal.pcbi.1006982)

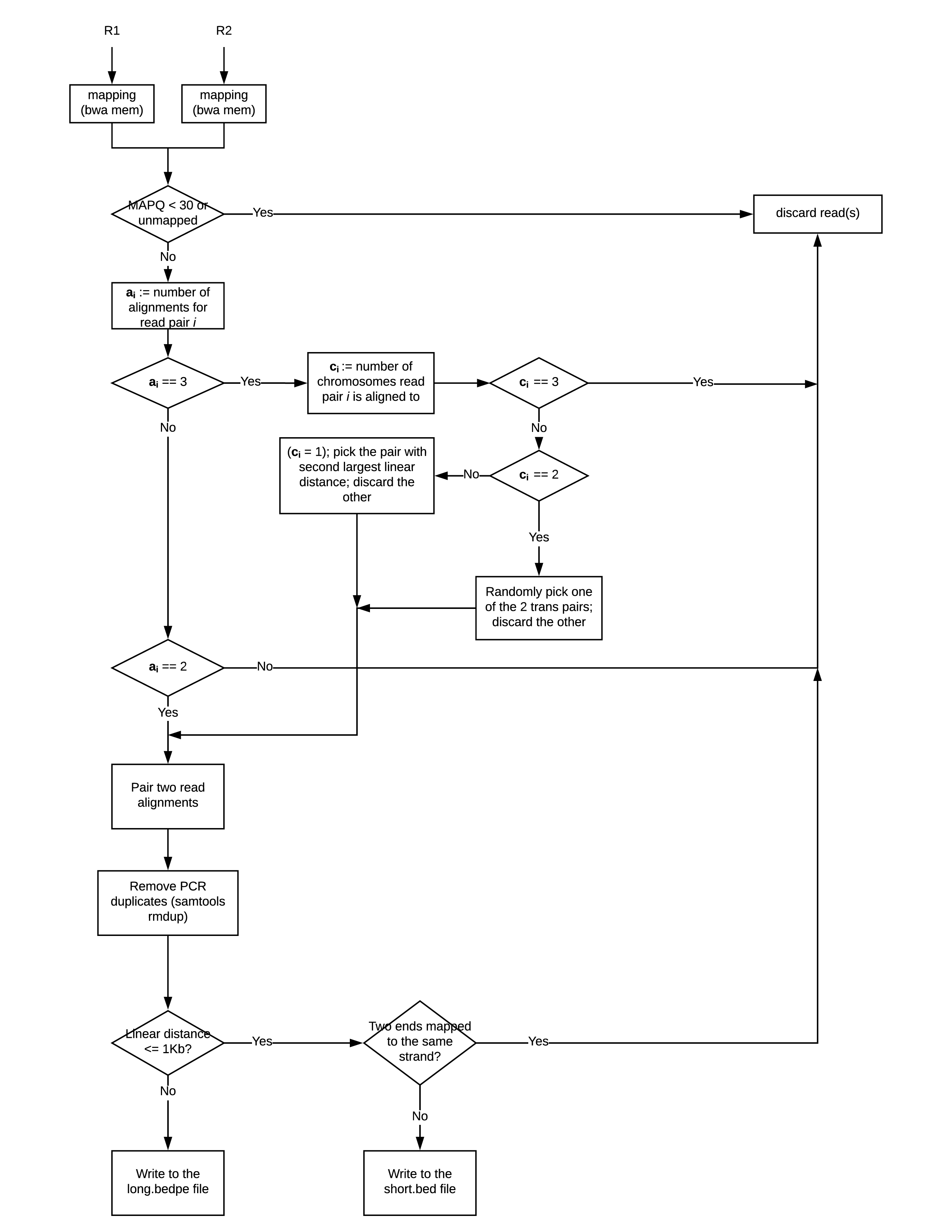

Supplement: S1 Fig — Details can be found in Methods section. (TIF) [file pcbi.1006982.s001.tif]

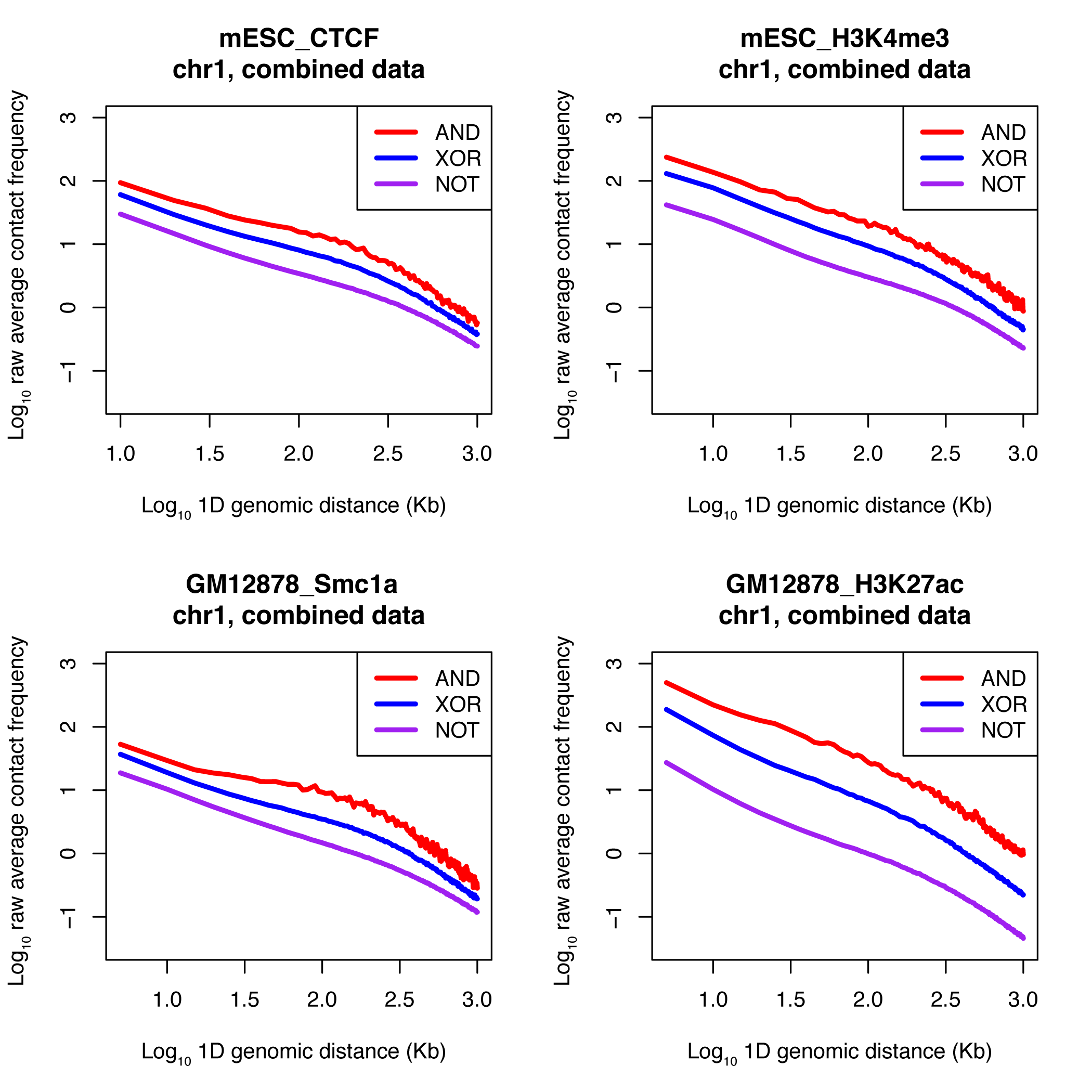

Supplement: S2 Fig — The results are based on combined datasets after merging two biological replicates; 10Kb resolution is used for mESC CTCF and 5Kb resolution is used for all the other datasets. The X-axis is Log10 genomic distance between two interacting bins (unit: Kb). The Y-axis is the Log10 average raw PLAC-seq/HiChIP contact frequency. The red line, blue and purple lines represent the contact probability for bin pairs in the “AND”, “XOR” and “NOT” set, respectively. (TIF) [file pcbi.1006982.s002.tif]

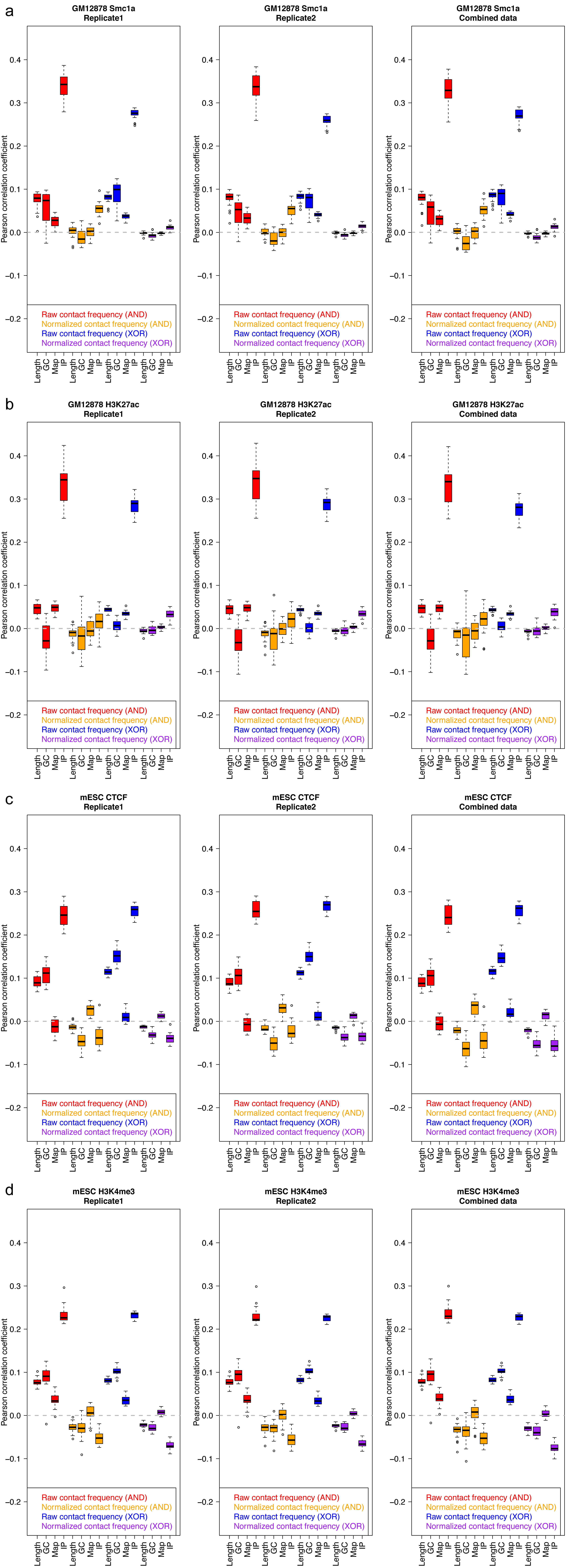

Supplement: S3 Fig — (a) MAPS removes biases in GM12878 Smc1a HiChIP data after normalization. For all autosomal chromosomes, we calculated the Pearson correlation coefficients (Y-axis) between the systemic biases (effective length, GC content, mappability, IP effect) and the raw contact frequency in the “AND” set, the normalized contact frequency in the “AND” set, the raw contact frequency in the “XOR” set and the normalized contact frequency in the “XOR” set, highlighted in red, yellow, blue and purple boxes, respectively. The grey dash line presents the Pearson correlation coefficient zero. Three panels show the results in replicate 1, replicate 2, and the combined data (replicate 1 + replicate 2), respectively. (b-d) Similar to S3 Fig a, MAPS removes biases in GM12878 H3K27ac HiChIP data (b), mESC CTCF PLAC-seq data (c) and mESC H3K4me3 PLAC-seq data (d). (TIF) [file pcbi.1006982.s003.tif]

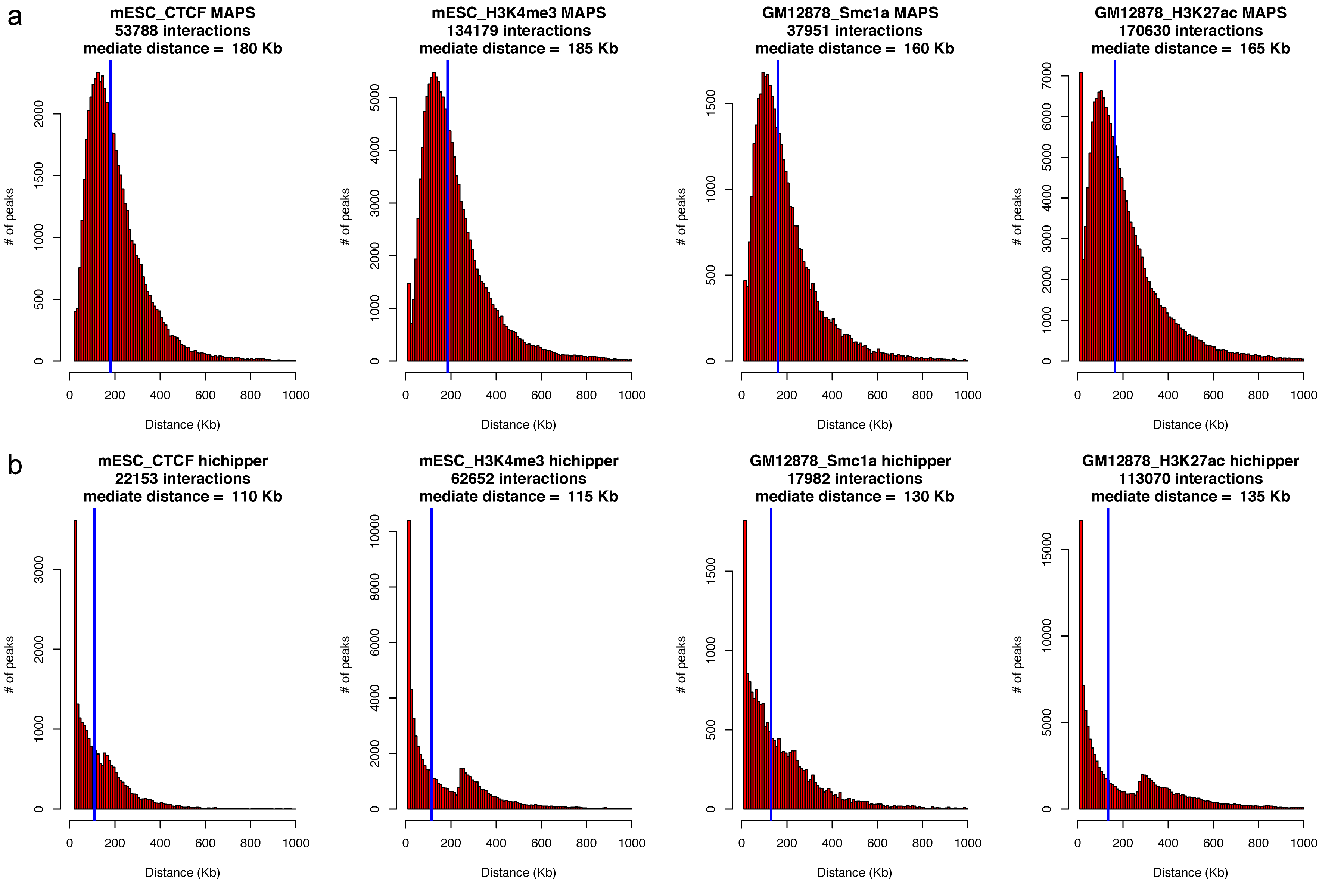

Supplement: S4 Fig — (a) The number of interactions and the distribution of interaction length of MAPS-identified interactions. From left to right are the results of MAPS calls from mESC CTCF PLAC-seq, mESC H3K4me3 PLAC-seq, GM12878 Smc1a HiChIP and GM12878 H3K27ac HiChIP combined data (replicate 1 + replicate 2). Each histogram shows the distribution of interaction length. The vertical blue bar represents the median distance of interactions. (b) Similar to S4 Fig a, the number of interactions and the distribution of interaction length of hichipper-identified interactions. (TIF) [file pcbi.1006982.s004.tif]

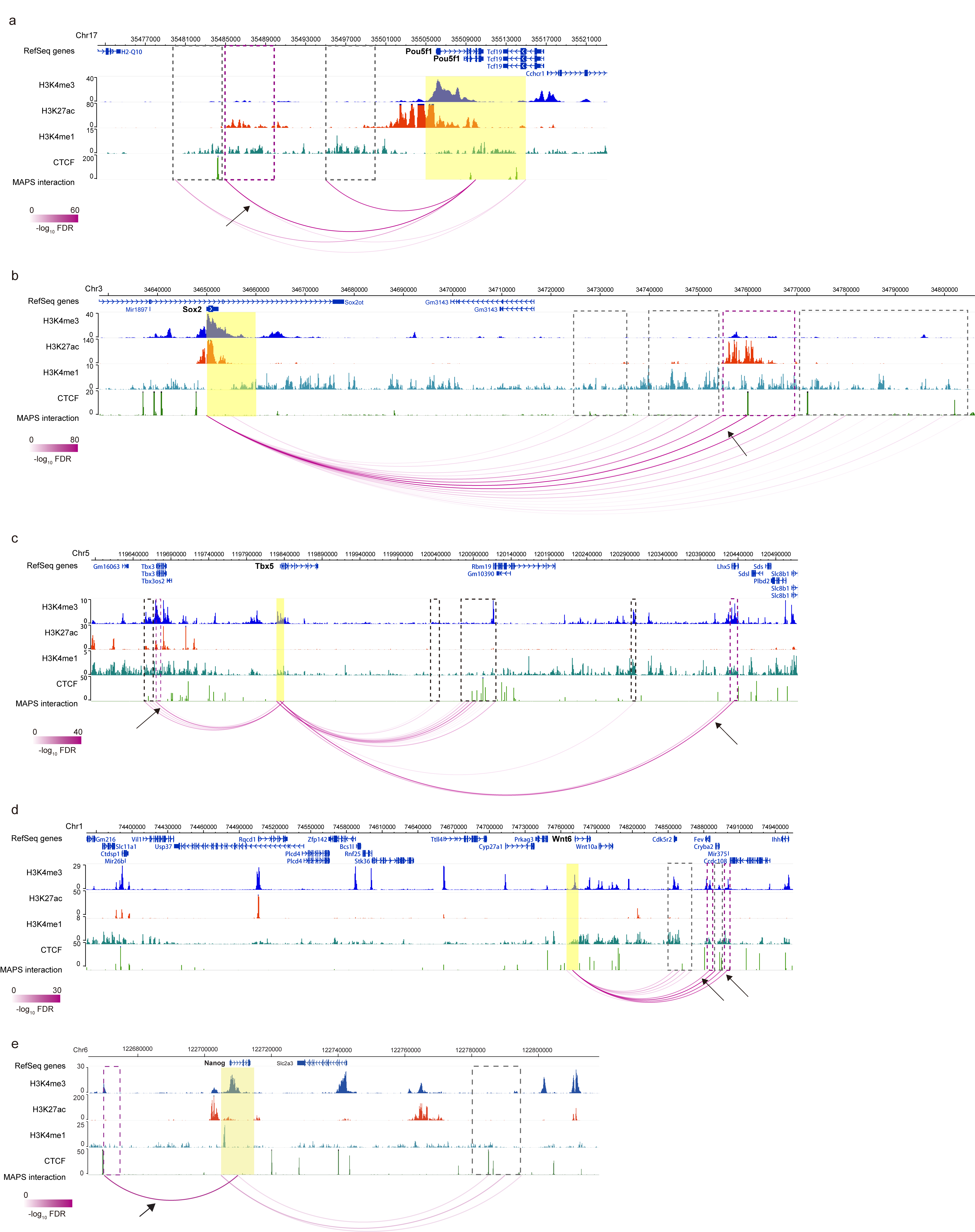

Supplement: S5 Fig — MAPS-identified interactions from mESC H3K4me3 PLAC-seq data anchored at: (a) Pou5f1 promoter, (b) Sox2 promoter, (c) Tbx5 promoter, (d) Wnt6 promoter, (e) Nanog promoter. Anchor regions around target promoter are highlighted by yellow boxes. The MAPS-identified interactions overlapping the anchor regions are marked by magenta arcs. The black arrow points to the interaction verified in previous publications [16–20] and the other end of the interaction is marked by magenta boxes. Additional interacting regions identified by MAPS are marked by grey boxes. (TIF) [file pcbi.1006982.s005.tif]

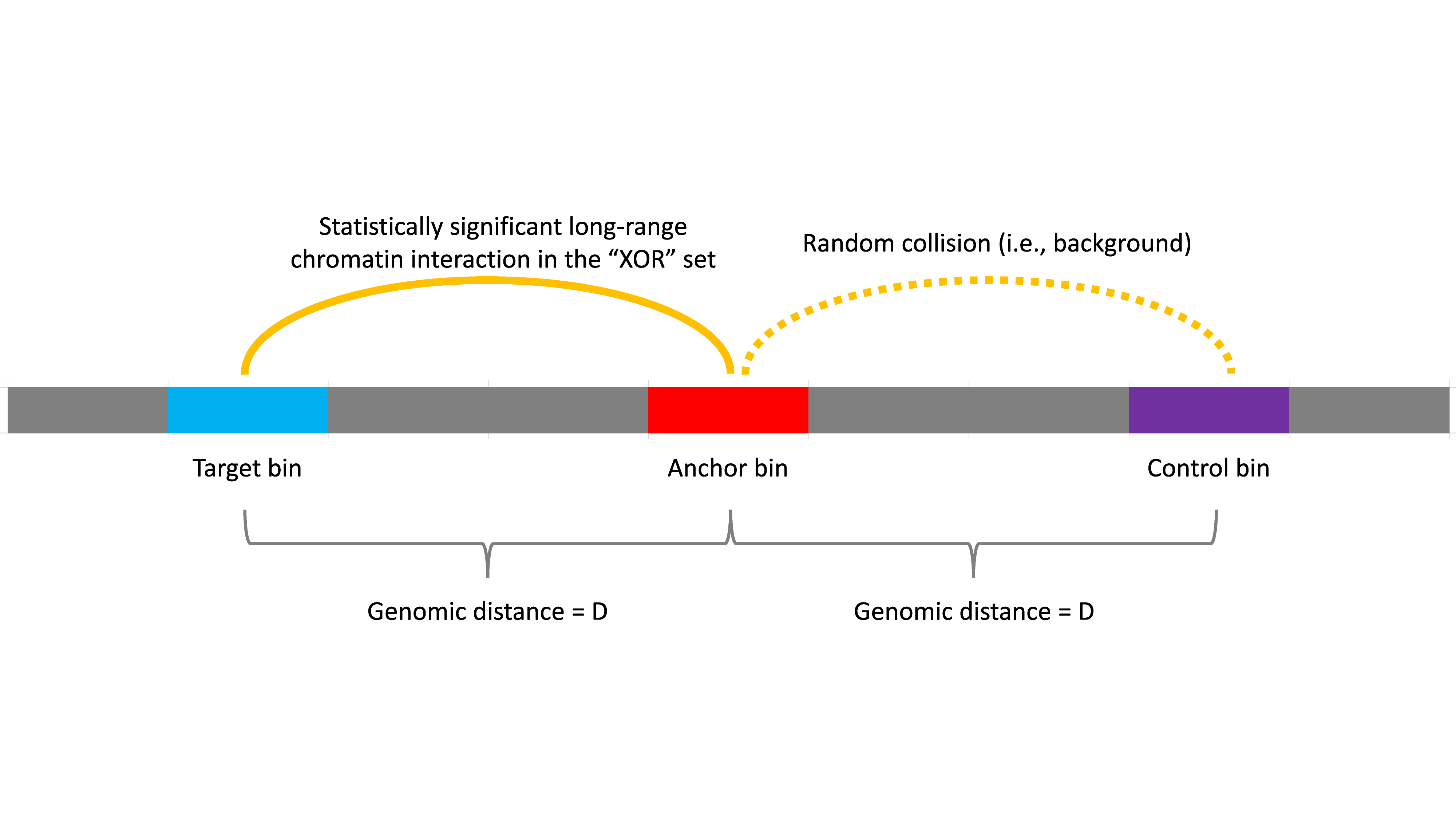

Supplement: S6 Fig — The solid yellow curve at left represents a statistically significant long-range chromatin interaction in the “XOR” set, connecting the anchor bin (the red box) and the target bin (the blue box). The dashed yellow curve at right represents a random collision between the anchor bin (the red box) and the control bin (the purple box). The interaction and the random collision has the same genomic distance. (TIF) [file pcbi.1006982.s006.tif]

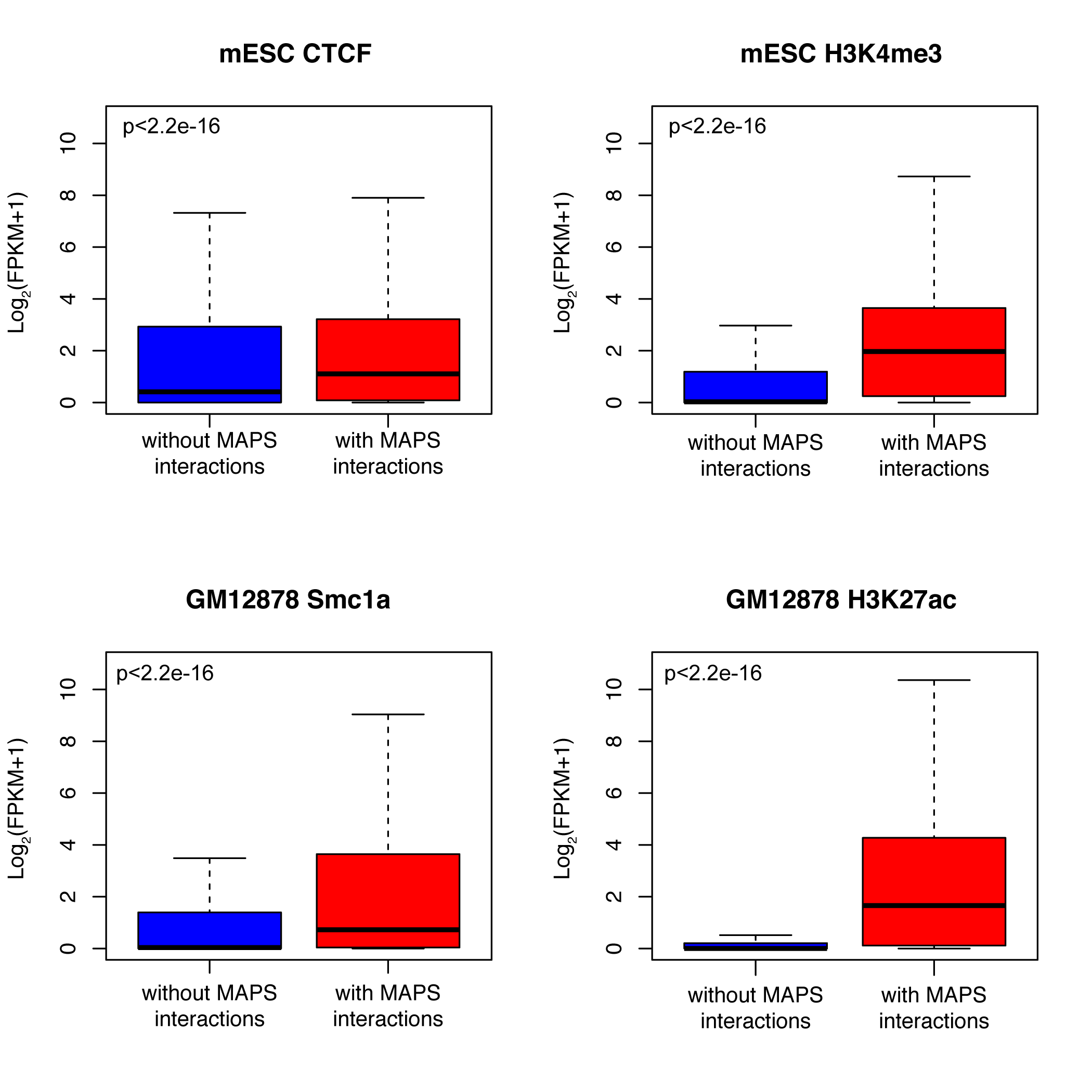

Supplement: S7 Fig — In each panel, the y-axis represents the log2(FPKM+1). The red box and blue box represent the genes in which TSSs are associated with MAPS-identified interactions and genes in which TSSs are not associated with MAPS-identified interactions, respectively. For all four datasets, genes in which TSS involves with MAPS-identified interactions have significantly higher expression than genes in which TSS does not involve with MAPS-identified interactions (p<2.2e-16). (TIF) [file pcbi.1006982.s007.tif]

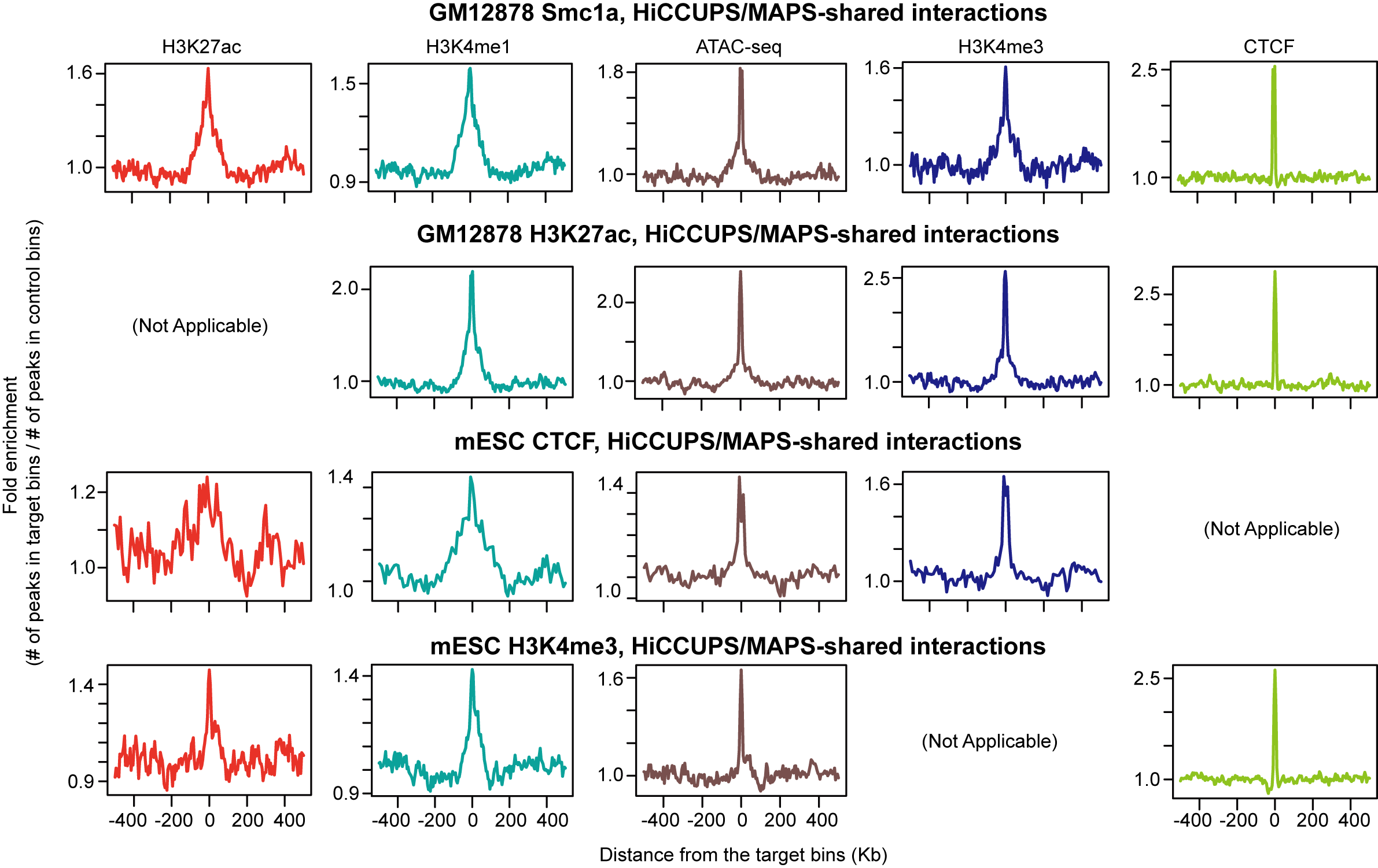

Supplement: S8 Fig — Enrichment of H3K27ac (ChIP-seq peaks), H3K4me1 (ChIP-seq peaks), ATAC-seq peaks, H3K4me3 (ChIP-seq peaks) and CTCF (ChIP-seq peaks) in a window of 500Kb around the target bins for all four datasets. Due to the definition of XOR set of interactions, H3K27ac, H3K4me3 and CTCF enrichment level is not analyzed for GM12878 H3K27ac HiChIP, mESC H3K4me3 and mESC CTCF PLAC-seq data, respectively. (TIF) [file pcbi.1006982.s008.tif]

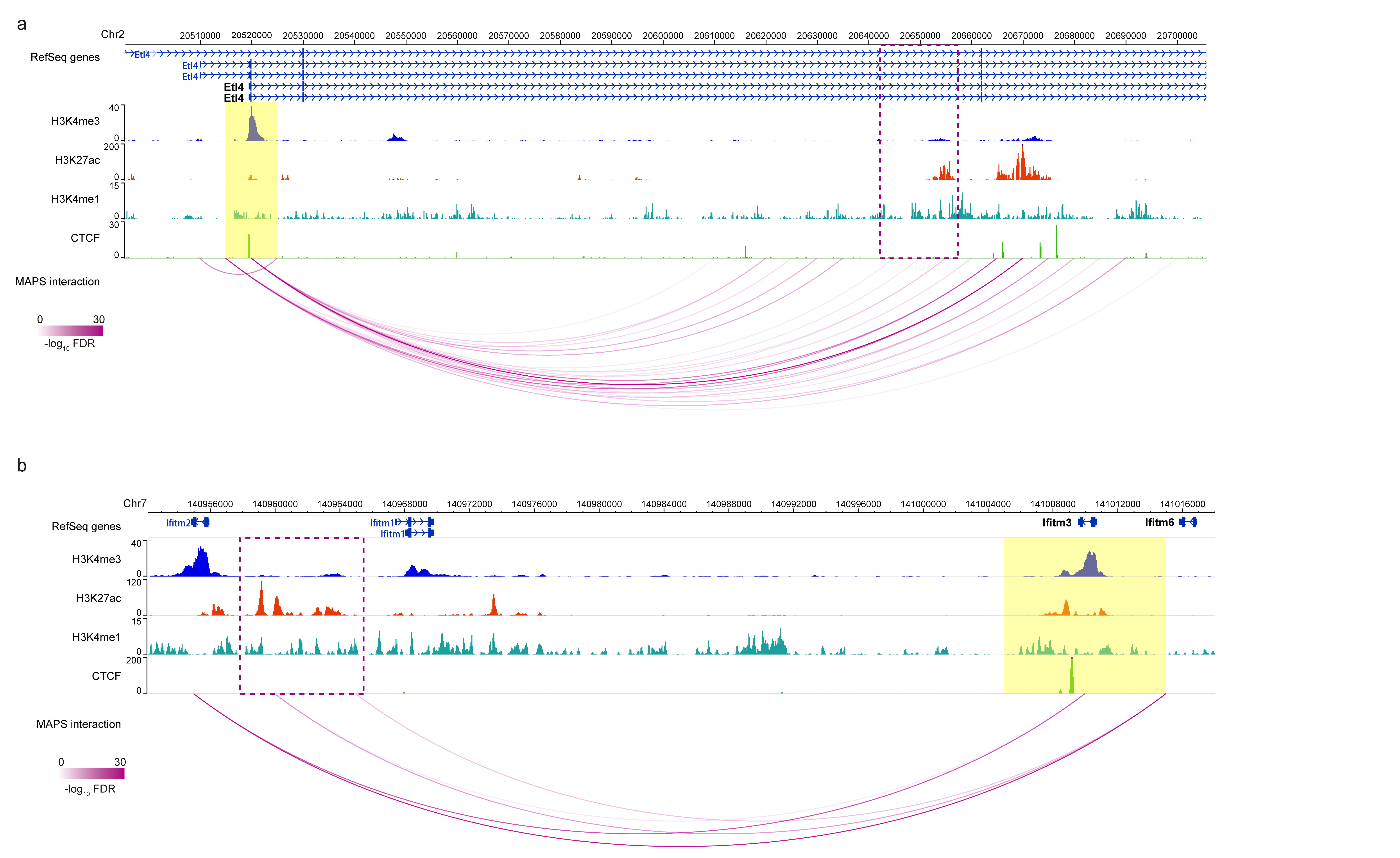

Supplement: S9 Fig — MAPS-identified interactions from mESC H3K4me3 PLAC-seq data anchored at: (a) Elt4 promoter (chr2:20,515,000–20,525,000), (b) Ifitm3 promoter (chr7:141,005,000–141,015,000). Anchor regions around target promoters are highlighted by yellow boxes. The MAPS-identified interactions overlapping this anchor region are marked by magenta arcs. The deleted enhancer regions in Moorthy et al study [24] are marked by magenta boxes. (TIF) [file pcbi.1006982.s009.tif]

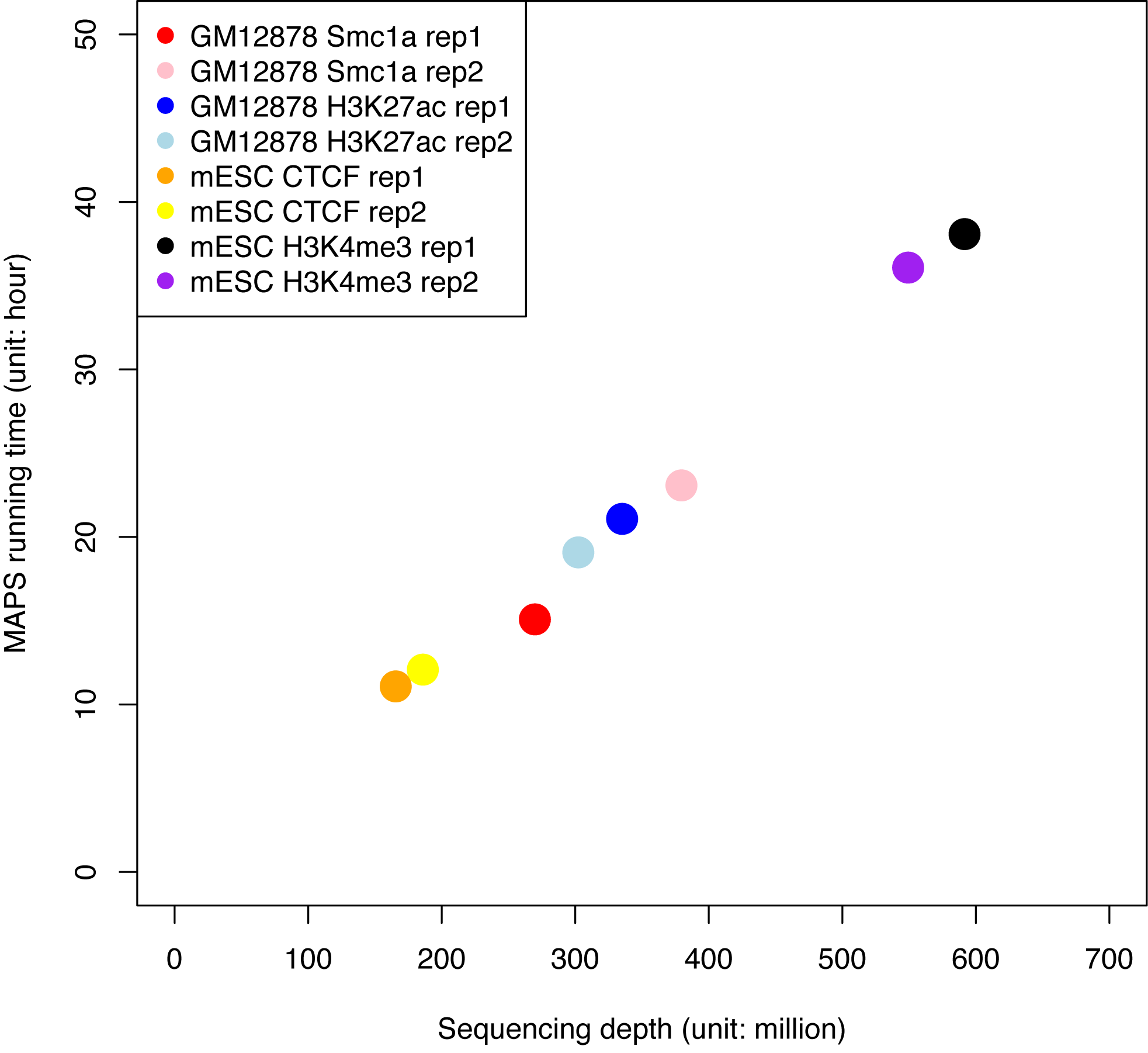

Supplement: S10 Fig — (TIF) [file pcbi.1006982.s010.tif]
