# Supplementary material for: MAPS: Model-based analysis of long-range chromatin interactions from PLAC-seq and HiChIP experiments
Source: PLoS Comput Biol. 2019 Apr 15;15(4):e1006982. doi: 10.1371/journal.pcbi.1006982 (PMC6483256; doi:10.1371/journal.pcbi.1006982)
Supplement: S1 Text — Note 1. Similarities and differences between PLAC-seq and HiChIP protocols. Note 2. Fit-Hi-C, HiCCUPS, Mango and hichipper are not optimal for the identification of long-range chromatin interactions from PLAC-seq and HiChIP data. Note 3. Justification of zero-truncated Poisson model used in MAPS. Note 4. MAPS analysis at finer resolution. Note 5. MAPS analysis with extended genomic distance range. Note 6. Justification of threshold values used in the MAPS interaction calling component. Note 7. Reproducibility of HiCCUPS loops. Note 8. Selecting HiCCUPS loops which are detectable in PLAC-seq and HiChIP data. Note 9. Detailed experimental procedures of PLAC-seq on F123 cells. (DOCX) [file pcbi.1006982.s026.docx]

**Supporting information**

**Note 1. Similarities and differences between PLAC-seq and HiChIP protocols.**

PLAC-seq and HiChIP share the same experimental design: both methods perform in situ proximity ligation prior to ChIP enrichment, thus enriching the ligation products with one or both ends bound by the protein of interest. The differences between these two methods are at chromatin shearing and library construction steps. PLAC-seq shears the chromatins into fragments with an average length of several hundred base pairs long for ChIP and then constructs libraries through end-repair, A-tailing and adapter ligations; HiChIP shears the chromatins into a few kilobase for ChIP and then constructs libraries using Tn5 transposase. Such technical difference is minor and does not change the property of PLAC-seq or HiChIP data. Therefore, the analysis pipeline developed for HiChIP data (such as hichipper) is expected to work comparably on PLAC-seq data and vice versa.

**Note 2.** **Fit-Hi-C, HiCCUPS, Mango and hichipper are not optimal for the identification of long-range chromatin interactions from PLAC-seq and HiChIP data.**

**(1) Fit-Hi-C (developed for Hi-C)**

Fit-Hi-C is developed for Hi-C to detect genome-wide long-range interactions. It adopts a global background model to estimate the expected contact frequency for each pair of genomic loci. It considers the systematic biases in Hi-C data but does not consider the immunoprecipitation bias, which is specific to PLAC-seq and HiChIP data. Fit-Hi-C treats all bin pairs in the NOT, XOR, AND set (defined in **Fig 1**) as equal and estimates their expected contact frequency in the same way. This is problematic since the NOT set should be excluded *a priori* and XOR, AND sets have significantly different levels of enrichment due to the nature of PLAC-seq and HiChIP technologies (**S2 Fig**).

**(2) HiCCUPS (developed for Hi-C)**

HiCCUPS is also developed for Hi-C but it defines significant interactions via a local background model by comparing the contact frequency in each bin pair with its surrounding regions. Same as Fit-Hi-C, HiCCUPS treats bin pairs from AND, XOR or NOT sets equally. HiCCUPS recommends the matrix balancing algorithm for data normalization, which is not suitable for PLAC-seq/HiChIP since bins with and without protein binding have different contact frequency, which violates the crucial “equal visibility” assumption implied by the matrix-balancing algorithms.

**(3) Mango (developed for ChIA-PET)**

Mango is developed for ChIA-PET, which also combines ChIP and proximity ligation. In ChIA-PET IP is performed prior to proximity ligation, whereas in PLAC-seq/HiChIP proximity ligation is performed first, leading to two major differences in their data analysis: 1) ChIA-PET can only detect interactions present in the AND set while PLAC-seq/HiChIP enables the detection of interactions in both AND and XOR sets since the ligated products having only one end bound by the target protein can also be enriched in PLAC-seq/HiChIP; 2) MACS2 application to PLAC-seq/HiChIP data results in high false positives (1) due to the restriction enzyme cutting bias and the lack of IP input control. Therefore, Mango can only detect interactions in the AND set and the result for the AND set is also suboptimal.

**(4) hichipper (developed for HiChIP)**

hichipper improves the identification of anchor regions for Mango implementation to better detect long-range interactions from the AND set. However, since hichipper still relies on Mango, it cannot detect interactions in the XOR set either. Applying hichipper to PLAC-seq/HiChIP data therefore results in relatively low sensitivity in detecting long-range chromatin interactions (**Fig 2**).

**Note 3. Justification of zero-truncated Poisson model used in MAPS.**

Our MAPS is built upon our previous work HiCNorm (2) but these two methods have distinct purposes. HiCNorm is designed to normalize raw Hi-C contact frequency matrix, therefore, it takes all bin pairs into consideration and models the raw count as a Poisson distribution. In contrast, MAPS is designed to identify statistically significant long-range chromatin interactions from PLAC-seq and HiChIP data at high resolution (kilobase level). Since the PLAC-seq and HiChIP data at high resolution (5Kb or 10Kb in our paper) are extremely sparse, MAPS only takes bin pairs with contact frequency >=1 into consideration. The Poisson distribution used in HiCNorm does not fit such zero-truncated count data, and it is a natural choice to use zero-truncated Poisson distribution instead. We used mESC H3K4me3 PLAC-seq data (combining two biological replicates) as an illustrative example to evaluate the model fitting, measured by the Akaike information criterion (AIC). Specifically, AIC of the Poisson and zero-truncated Poisson model are 50,505,722.01 and 45,877,879.29, respectively. Since lower AIC suggests better model fitting, our results demonstrate that zero-truncated Poisson model used in MAPS fits PLAC-seq data better than the Poisson model used in HiCNorm.

**Note 4. MAPS analysis at finer resolution.**

To evaluate MAPS performance at finer resolution, we applied MAPS to the deeply sequenced mESC H3K4me3 PLAC-seq data (combining two biological replicates) at 2Kb resolution while keeping all the other parameters the same. We identified 63,323 interactions in total, which is fewer than the number of interactions identified at 5Kb resolution (134,179 interactions) since the same threshold values are used to define significant interactions. The interactions identified at different resolutions are highly overlapped: 50,798 out of 63,323 (80.2%) 2Kb interactions can be captured by 5Kb interactions and 68,980 out of 134,179 (51.4%) 5Kb interactions can be captured by 2Kb interactions (with the same definition used in the “**Reproducibility analysis**” section, see **Methods**). We then evaluated the sensitivity of these 2Kb interactions. Since there is no publicly available 2Kb bin resolution HiCCUPS loops, we still used the 1,989 testable HiCCUPS loops at 5Kb bin resolution for comparison (**S6 Table**). We found that 1,262 out of all 1,989 testable HiCCUPS loops (63.4%) are captured by MAPS at 2Kb resolution, suggesting that MAPS can still achieve relatively high sensitivity at finer bin resolution. We also checked the CTCF motif orientations of these 2Kb interactions and found the convergent CTCF motif rate of testable interactions is 55.8%, which is comparable with that of 5Kb interactions (53.3%). Taken together, the results indicate that MAPS also works well at finer resolution despite some sacrifice in sensitivity due to the lower average read counts in each bin pair.

**Note 5. MAPS analysis with extended genomic distance range.**

In theory MAPS can be applied to detect intra-chromosomal interactions of any genomic distance depending on the user’s preference and we chose to consider interactions within 1Mb for two reasons: 1) most interactions happen in the same TADs (within mega-base scale); 2) from the distribution of MAPS call within 1Mb (**S4 Fig**), we expected to detect few new interactions even after extending to a longer distance given the sequencing depth of the PLAC-seq/HiChIP datasets. To evaluate the performance of MAPS with extended genomic distance range, we applied MAPS to the deeply sequenced mESC H3K4me3 PLAC-seq data (combining two biological replicates) at 5Kb bin resolution searching for long-range interaction within genomic distance of 2Mb. We found in total 131,063 interactions using the same thresholds described in the paper and 1,178 of them (0.9%) are between 1Mb and 2Mb. To evaluate the sensitivity, we selected testable HiCCUPS loops between 1Mb and 2Mb from deeply sequenced mESC in situ Hi-C data using the same filtering as described in **Note 8 in S1 Text**, and obtained 35 testable HiCCUPS loops (**S6 Table**). All of these 35 HiCCUPS loops are captured by MAPS. We further checked their CTCF motif orientations and found that the convergent CTCF motif rate of testable interactions is 47.5%, which is slightly lower than that of MAPS-identified interactions within 1Mb (53.3%). Taken together, above results suggest that MAPS performs almost equally well after extending to a longer genomic distance (i.e., 2Mb range).

**Note 6. Justification of threshold values used in the MAPS interaction calling component.**

To select the appropriate threshold used in the MAPS interaction calling component, we examined HiCCUPS loops identified from deeply sequenced in situ Hi-C data, which have been widely accepted as a confident list of long-range chromatin interactions with low false positive rate. We used the HiCCUPS loops from both GM12878 in situ Hi-C data (~4.9 billion reads) reported by Rao et al. study (3) and mESC in situ Hi-C data (~7.3 billion reads) (4) (**S5 Table**). First of all, we found that HiCCUPS loops have minimal raw contact frequency of 12 or 13 for both human and mouse data, and for both 5Kb and 10Kb bin resolution. Therefore, we set up minimal raw contact frequency as 12 in MAPS. In addition, HiCCUPS uses four local background models, including horizontal, vertical, lower-left and donut. For each HiCCUPS loop, we define the normalized contact frequency as the ratio between the observed contact frequency and the maximal value of four local background models. We found that the minimal normalized contact frequency is around 1.5 across all 4 testing datasets. In addition, all HiCCUPS loops have FDR < 10%. Based on the threshold values used in HiCCUPS loops, in MAPS, we set up a more stringent minimal normalized contact frequency 2, and use a more stringent threshold FDR < 1% to ensure low false positive rate.

**Note 7. Reproducibility of HiCCUPS loops.**

The Figure 3B in Rao et al study (3) shows the reproducibility of HiCCUPS loops at 10Kb bin resolution. Between 8,054 loops identified from the primary dataset and 7,484 loops identified from the replicate data, 5,403 loops are shared. Therefore, the reproducibility of loops in the primary and replicate dataset is 67.1% and 72.2%, respectively.

To make a fair comparison with the reproducibility analysis performed for PLAC-seq and HiChIP data, we selected 7,393 and 7,055 loops which are within 1Mb in the autosomal chromosomes, for the primary and replicate dataset, respectively. Next, we defined a loop ($i,j$) in one dataset is reproducible in the other dataset, if and only if there exists a loop ($m,n$) in the other dataset such as $\max\left\{ d_{im},d_{jn} \right\}\leq$ 15Kb. Here $d_{ij}$ is the genomic distance between the center of bin $i$ and the center of bin $j$. Based on such definition, 4,752 out of 7,393 loops (64.3%) in the primary dataset is reproducible in the replicate dataset, while 4,753 out of 7,055 loops (67.4%) in the replicate dataset is reproducible in the primary dataset.

**Note 8. Selecting HiCCUPS loops which are detectable in PLAC-seq and HiChIP data.**

We applied the following five filters to select HiCCUPS loops which are detectable in PLAC-seq and HiChIP data (**S6 Table**). (1) We first selected loops in autosomal chromosomes. (2) We then kept loops with genomic distance within 1Mb. (3) We only kept loops in which at least one of two interacting bins binds to the protein of interest (i.e., the “AND” set and the “XOR” set). (4) We noticed that some HiCCUPS loops identified from the deeply sequenced in situ Hi-C data have low raw contact frequency in PLAC-seq and HiChIP data, therefore they are not detected by either MAPS or hichipper. To account for substantial difference in sequencing depth between in situ Hi-C data and PLAC-seq and HiChIP data, we only kept loops with raw contact frequency in PLAC-seq and HiChIP data >= 12. Note that 12 is the minimal raw contact frequency for both HiCCUPS and MAPS (**S5 Table**). (5) The default FDR threshold for HiCCUPS, MAPS and hichipper is 10%, 1% (0.01% for singletons) and 1% (0.01% for singletons), respectively. To make a fair comparison in the sensitivity analysis, we only kept HiCCUPS loops with FDR < 1%.

**Note 9. Detailed experimental procedures of PLAC-seq on F123 cells.**

**Day 1: In situ digestion and proximity ligation** (note**:** NO stop point in **Day 1** from **Step 1-27**)

Buffer:

Lysis buffer: 10 mM Tris•HCl (pH 8.0), 10 mM NaCl, 0.2% IGEPAL CA-630. Store at 4°C; add proteinase inhibitor cocktail (PIC) before use.

**Cell lysis and MboI digestion**

1. Resuspend 1-3 million crosslinked F123 cells in 300 μl cold lysis buffer with PIC and incubate on ice for at least 15 minutes (1 million for H3K4me3 PLAC-seq and 3 million for CTCF PLAC-seq).
2. Spin at 2500×*g* for 5 minutes at 4°C and remove the supernatant.
3. Wash the pellet with 500 μl cold lysis buffer with PIC. Spin at 2500×*g* for 5 minutes at 4°C and then remove the supernatant.
4. Gently resuspend cell pellet in 50 μl 0.5% SDS and incubate for exactly 10 minutes at 62°C.
5. Add 135 μl water and 25 μl freshly prepared 10% Triton X-100 to quench the SDS. Mix well and incubate for 15 minutes at 37°C.
6. Add 25 μl 10×NEBuffer 2 and 4 μl of MboI (25 U/μl). Mix well and digest chromatin for **exactly 2 hours** at 37°C in thermomixer, shaking at 900 rpm.

**End fill-in and proximity ligation**

1. Inactivate MboI by incubation for 20 minutes at 62°C.
2. Cool the reaction to room temperature (takes ~10 minutes).
3. Add the following reagents to fill in overhangs and mark with biotin:

| **Component** | **Amount (μl)** |
| --- | --- |
| dCTP (10 mM) | 1.5 |
| dGTP (10 mM) | 1.5 |
| dTTP (10 mM) | 1.5 |
| Biotin-14-dATP (0.4 mM) | 37.5 |
| Klenow (5 U/μl) | 8 |
| Total | 50 |

1. Mix well and incubate for 1 hour at 37°C in thermomixer, shaking at 900 rpm.
2. Prepare ligation master mix as follows:

| **Component** | **Amount (μl)** |
| --- | --- |
| water | 664 |
| 10×T4 ligase buffer (NEB) | 120 |
| 10% Triton X-100 | 100 |
| BSA (20 mg/ml) | 6 |
| T4 DNA ligase (NEB, 400 U/μl) | 10 |
| Total | 900 |

1. Add above ligase master mix and mix well by inverting the tube. Rotate at room temperature for 2 hours.

**II. ChIP (day 1 and day 2)**

Regents and buffers:

Protein G Sepharose 4 Fast Flow from GE

RIPA buffer: 10 mM Tris•HCl (pH 8.0), 140 mM NaCl, 1 mM EDTA, 1% Triton X-100, 0.1% SDS, 0.1% sodium deoxycholate. Store at 4°C; add proteinase inhibitor cocktail (PIC) before use.

high-salt RIPA buffer: 10 mM Tris•HCl (pH 8.0), 300 mM NaCl, 1 mM EDTA, 1% Triton X-100, 0.1% SDS, 0.1% sodium deoxycholate. Store at 4°C.

LiCl buffer: 10 mM Tris•HCl (pH 8.0), 150 mM LiCl, 1 mM EDTA, 0.5% IGEPAL CA-630, 0.1% sodium deoxycholate. Store at 4°C.

TE buffer: 10 mM Tris•HCl (pH 8.0), 0.1 mM EDTA. Store at 4°C.

Extraction buffer: Add 15 μl of 10% SDS, 12 μl 5M NaCl and 1 μl RNase A (10 mg/mL) to 135 μl TE buffer before use.

**Preparation of sepharose beads (day 1)**

1. Transfer 30×2 μl beads per sample to two 1.7 mL microcentrifuge tubes.
2. Add 300×2 μl water to each tube to dilute the ethanol.
3. Spin at 400×*g* for 2 minutes and discard supernatant.
4. For cell lysate pre-clearing portion, resuspend in 500 μl RIPA buffer with PIC; for binding portion, resuspend 500 μl 0.5% BSA in RIPA buffer and rotate at 4°C overnight.

**Chromatin fragmentation and Immunoprecipitation (day 1)**

1. After ligation, spin the nuclei (from **Step 12**) at 2500×*g* for 5 minutes at 4°C and discard supernatant.
2. Resuspend the pellet in 120 μl RIPA buffer with PIC and incubate on ice for 15 minutes.
3. Transfer the cell lysate to microTUBE AFA Fiber Snap-Cap (PN 520045, Covaris) and shear the chromatin using Covaris M220 with the following setting: power, 75 W; duty factor, 10%; cycle per burst, 200; time, 10 minutes; temp, 7°C. Target size is 200-700 bp.
4. Transfer sheared chromatin to pre-chilled 1.7 ml microcentrifuge tube.
5. Wash the microTUBE with 100 μl RIPA buffer with PIC and add to the sample. Add additional 300 ul RIPA buffer with PIC to bring the volume to 500 μl.
6. Spin at 15,000×*g* for 15 minutes at 4°C to clear the cell lysate and transfer the supernatant to a pre-chilled, clean 1.7 ml microcentrifuge tube.
7. Mix the supernatant with washed beads from **Step 16**, pre-clearing portion and rotate for 3 hours at 4°C.
8. After pre-clearing, spin down the beads at 400×*g* for 2 minutes at 4°C.
9. Transfer the pre-cleared cell lysate to a pre-chilled, clean 1.7 ml microcentrifuge tube. Transfer 50 μl supernatant to another clean 1.7 ml microcentrifuge tube and save as input (store at -20°C and continue from **Step 32**).
10. Add 5 μl H3K4me3 (cat.no. 04-745, Millipore) or 5 μg CTCF antibody (cat.no. sc-15914X, Santa Cruz) to the rest pre-cleared cell lysate and rotate at 4°C overnight.

**Binding, wash, reverse crosslinking and DNA purification (day 2)**

1. Take out the pre-blocked beads from **Step 16** (binding portion) and spin down the beads at 400×*g* for 2 minutes at 4°C. Discard the supernatant.
2. Add the mixture of cell lysate and antibody (from **Step 26**) to the pre-blocked beads and rotate at 4°C for 3 hours.
3. After binding, spin down the beads at 400×*g* for 2 minutes at 4°C. Discard the supernatant.
4. Resuspend beads in 1 mL RIPA buffer and shake at 1,000 rpm for 3 minutes at 4°C in thermomixer.
5. Spin down the beads at 400×*g* for 2 minutes at 4°C. Discard the supernatant.
6. Repeat the wash (**Step 30-31**) twice with RIPA buffer, twice with high-salt RIPA buffer, once with LiCl buffer and twice with TE buffer.
7. After final wash, add 163 μL extraction buffer to the beads and mix well, followed by shaking at 1,000 rpm for 1h at 37°C.
8. Add 20 μg proteinase K to each sample and shake at 1,000 rpm for at least 2 hours at 65°C.
9. Remove the tube from thermomixer and spin down the beads at 400×*g* for 2 minutes.
10. Transfer the supernatant to another clean 1.7 ml microcentrifuge tube and purify the ChIPed DNA with DNA Clean & Concentrator™-5 (Zymo, cat. no. D4014). Elute DNA with 25 μL 10mM Tris (pH 8.0) twice (total 50 μL).

**III. Library construction (day 3)**

Regents and buffers:

Magnetic beads: Dynabeads™ MyOne™ Streptavidin T1 or C1 (Thermo Scientific, cat. no. 65601 or 65001)

1×Tween Washing Buffer (TWB): 5 mM Tris-HCl (pH 7.5), 0.5 mM EDTA, 1M NaCl, 0.05% Tween 20

2× Binding Buffer (BB): 10 mM Tris-HCl (pH 7.5), 1 mM EDTA, 2M NaCl

**Biotin pull-down and adding sequencing adapters**

1. Wash 25 μL of T1 or C1 beads per sample twice with 400 μL TWB as follows:
   1. Resuspend beads in buffer.
   2. Incubate at RT for 3 min with rotation.
   3. Collect beads on magnet for 1 min.
   4. Remove supernatant.
2. Resuspend beads in 50 μL of BB.
3. Combine ChIPed DNA (50 μL from **Step 36**) with resuspended beads.
4. Incubate sample at RT for 15 min with rotation.
5. Collect beads on magnet (1-2 min), and remove supernatant.
6. Wash beads twice with 500 μl of TWB as follows:
   1. Resuspend beads in buffer.
   2. Incubate for 2 min at 55°C in thermomixer, shaking at 900 rpm.
   3. Collect beads on magnet for 1 min.
   4. Discard supernatant.
7. Wash beads once with 100 μl 1×NEB T4 DNA ligase buffer as follows:
   1. Resuspend beads in buffer.
   2. Transfer to a new 1.7 ml microcentrifuge tube.
   3. Collect beads on magnet for 1 min.
   4. Discard supernatant.
8. Resuspend beads in 100 μL of master mix as follow (**End repair and removal of biotin from free ends**):

| **Component** | **Amount (μl)** |
| --- | --- |
| 1×NEB T4 DNA ligase buffer | 88 |
| dNTPs (25 mM each) | 2 |
| T4 DNA Polymerase (3 U/μl) | 4 |
| T4 Polynucleotide Kinase (10 U/μl) | 5 |
| Klenow (5 U/μl) | 1 |
| Total | 100 |

1. Incubate for 30 min at 24°C (RT) in thermomixer, shaking at 900 rpm.
2. Add 300 μl of TWB, collect beads on magnet (1 min), and remove supernatant.
3. Wash beads twice with 500 μl of TWB as **Step 42**.
4. Wash beads once with 100 μl 1×NEBuffer 2 as **Step 43**.
5. Resuspend beads in 100 μL of master mix as follow (**A-tailing**)

| **Component** | **Amount (μl)** |
| --- | --- |
| 1×NEBuffer 2 | 90 |
| dATPs (10 mM) | 5 |
| Klenow (3'-->5' exo-) (5 U/μl) | 5 |
| Total | 100 |

1. Incubate for 30 min at 37°C in thermomixer, shaking at 900 rpm.
2. Add 300 μl of TWB, collect beads on magnet (1 min), and remove supernatant.
3. Wash beads twice with 500 μl of TWB as **Step 42**.
4. Wash beads once with 100 μl 1× Quick Ligation Reaction Buffer as **Step 43**.
5. Resuspend beads in 50 μL 1× Quick Ligation Reaction Buffer.
6. Add the following to ligate illumina adapters:
   1. 3 μl of 1:10 diluted illumina adapter (TruSeq LT kit)
   2. 2 μl of Quick ligase (NEB).
7. Mix thoroughly and incubate for 15 min at RT with rotation.
8. Add 300 μl of TWB, collect beads on magnet (1 min), and remove supernatant.
9. Wash beads twice with 500 μl of TWB as **Step 42**.
10. Wash beads once with 100 μl 10 mM Tris-HCl as **Step 43**.
11. Resuspend beads in 45 μl 10 mM Tris-HCl (pH 8.0).

**Determination of cycle number using qPCR**

1. Prepare 1:1000 dilution of each sample (from **Step 60**) with 10 mM Tris-HCl (pH 8.0) as template for qPCR.
2. Set up qPCR using KAPA Library Quantication Kit (Illumina® platforms). Prepare triplicates per sample and DNA standards (1-6).
3. Calculate the cycle number needed to yield a final library concentration of 10-20 nM in 50 μl; 12 cycles for H3K4me3 PLAC-seq sample and 13 cycles for CTCF PLAC-seq sample.

**Final amplification and library purification**

1. After determination of optimal cycle number, amplify the libraries in 100 μl reaction using Illumina primers and protocol.

| **Component** | **Amount (μl)** |
| --- | --- |
| Template (from **Step 60**) | 44 |
| Mixed F/R primers (10 mM each) | 6 |
| KAPA HiFi HotStart ReadyMix (2×) | 50 |
| Total | 100 |

**Cycling protocol**:

| Initial denaturation | 95 ^o^C | 5 min |
| --- | --- | --- |
| Denaturation | 98 ^o^C | 20 s |
| Annealing | 65 ^o^C | 30 s |
| Extension | 72 ^o^C | 1 min |
|  | 12-13 cycles (determined in **Step 63**) | |
| Final extension | 72 ^o^C | 7 min |

Primer sequences:

Forward: 5'-AATGATACGGCGACCACCGAGATCTACAC

Reverse: 5'-CAAGCAGAAGACGGCATACGAGAT

1. Add 100 μl water to a total volume of 200 μl per sample.
2. Add 100 μl of AMPure XP beads (warm to RT before use) and mix well with pipette.
3. Incubate for 8 min at RT.
4. Collect beads on magnet for 2-5 min or until the solution is clear.
5. Transfer the supernatant to a new 1.7 ml microcentrifuge and then add 60 μl of AMPure XP beads.
6. Incubate for 8 min at RT.
7. Collect beads on magnet for 2-5 min or until the solution is clear.
8. Remove supernatant, and wash beads twice with freshly prepared 70% EtOH as follows:
   1. Add 1 ml 70% EtOH without disturb the beads.
   2. Incubate for 1 min on the magnetic stand.
   3. Remove supernatant.
9. Air dry for 5-8 min.
10. Elute in 50 μl 10 mM Tris-HCl (pH 8.0) as follows:
    1. Add 50 μl 10 mM Tris-HCl (pH 8.0)
    2. Mix well with pipette
    3. Incubate at RT for 10 min.
11. Collect beads on magnet for 2 min.
12. Transfer supernatant (containing final library) to new tube.
13. Use 2 μl for Qubit dsDNA HS quantification and then dilute to appropriate concentration for Tapestation to check the size distribution.

**References**

1. Lareau CA, Aryee MJ. hichipper: a preprocessing pipeline for calling DNA loops from HiChIP data. Nature methods. 2018;15(3):155-6.

2. Hu M, Deng K, Selvaraj S, Qin Z, Ren B, Liu JS. HiCNorm: removing biases in Hi-C data via Poisson regression. Bioinformatics (Oxford, England). 2012;28(23):3131-3.

3. Rao Suhas SP, Huntley Miriam H, Durand Neva C, Stamenova Elena K, Bochkov Ivan D, Robinson James T, et al. A 3D Map of the Human Genome at Kilobase Resolution Reveals Principles of Chromatin Looping. Cell. 2014;159(7):1665-80.

4. Bonev B, Mendelson Cohen N, Szabo Q, Fritsch L, Papadopoulos GL, Lubling Y, et al. Multiscale 3D Genome Rewiring during Mouse Neural Development. Cell. 2017;171(3):557-72.e24.
